# Supplementary material for: Scientific Literature Database Coverage of Randomized Clinical Trials for Central Serous Chorioretinopathy
Source: J Pers Med. 2023 Jun 12;13(6):983. doi: 10.3390/jpm13060983 (PMC10305376; doi:10.3390/jpm13060983)
Supplement: Supplementary file 1 [file jpm-13-00983-s001.zip › Supplementary File S1.pdf]

Supplementary File S1. Documentation of the search in the individual databases.

BIOSIS Previews:

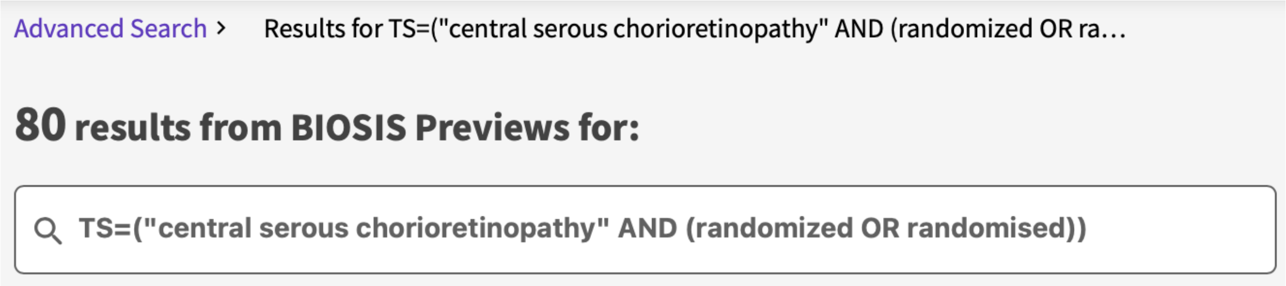

CINAHL:

Search History/Alerts

Print Search History   Retrieve Searches   Retrieve Alerts   Save Searches / Alerts

Select / deselect all | Search with AND | Search with OR | Delete Searches | Refresh Search Results

Search ID#	Search Terms	Search Options	Actions
S1	"central serous chorioretinopathy" AND (randomized OR randomised)	**Expanders** - Apply equivalent subjects<br>**Search modes** - Boolean/Phrase	View Results (26) View Details Edit

Cochrane Central:

141 Trials matching **"central serous chorioretinopathy" AND (randomized OR randomised) in Title Abstract Keyword - (Word variations have been searched)**

Cochrane Central Register of Controlled Trials
Issue 4 of 12, April 2023

Current Contents Connect:

Advanced Search › Results for TS=("central serous chorioretinopathy" AND (randomized OR ra…

97 results from Current Contents Connect for:

TS=("central serous chorioretinopathy" AND (randomized OR randomised))

Data Citation Index:

Search in: **Data Citation Index** Editions: **All**

Your search found no results

Check the spelling and/or broaden your search parameters

0/1 Combine Sets Export

☐	1	TS=("central serous chorioretinopathy" AND (randomized OR randomised))	0

Derwent Innovations Index:

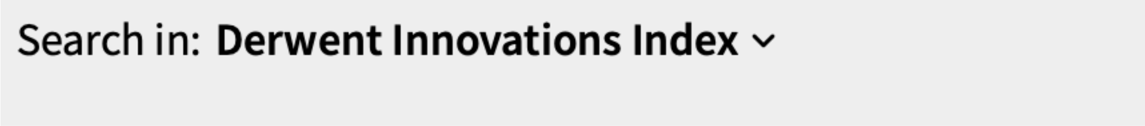

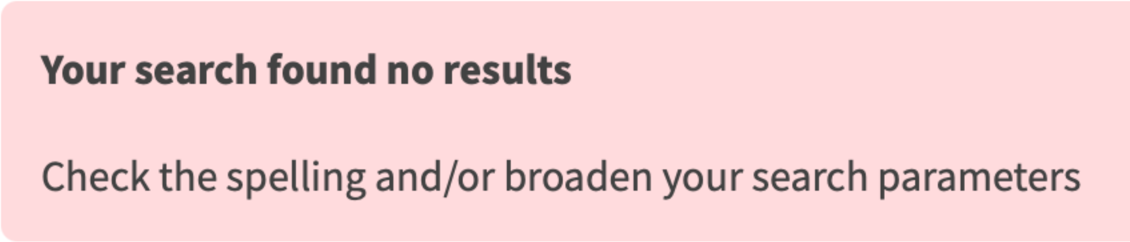

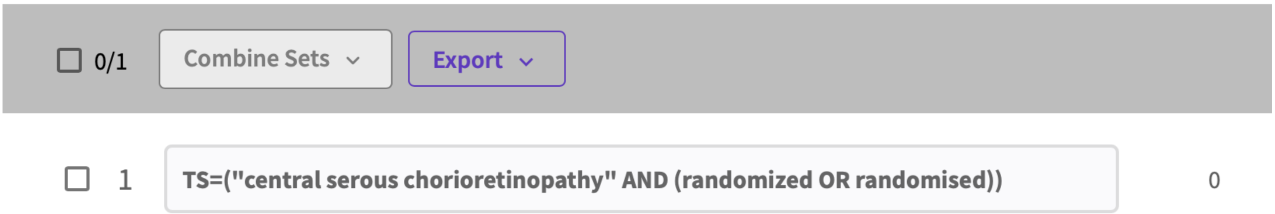

EMBASE:

▼ Search History (1) View Saved

	# ▲	Searches	Results	Type	Actions	Annotations
	1	("central serous chorioretinopathy" and (randomized or randomised)).mp. [mp=title, abstract, heading word, drug trade name, original title, device manufacturer, drug manufacturer, device trade name, keyword heading word, floating subheading word, candidate term word]	184	Advanced	Display Results | More	

Save Remove Combine with: AND OR

KCI-Korean Journal Database:

Advanced Search > Results for TS=("central serous chorioretinopathy" AND (randomized OR ra…

4 results from KCI-Korean Journal Database for:

TS=("central serous chorioretinopathy" AND (randomized OR randomised))

MEDLINE:

Advanced Search > Results for TS=("central serous chorioretinopathy" AND (randomized OR ra…

102 results from MEDLINE® for:

TS=("central serous chorioretinopathy" AND (randomized OR randomised))

PubMed:

History and Search Details

Search	Actions	Details	Query	Results	Time
#1			Search: **"central serous chorioretinopathy" AND (randomized OR randomised)** Sort by: **Most Recent** "central serous chorioretinopathy"[All Fields] AND ("random allocation"[MeSH Terms] OR ("random"[All Fields] AND "allocation"[All Fields]) OR "random allocation"[All Fields] OR "randomization"[All Fields] OR "randomized"[All Fields] OR "random"[All Fields] OR "randomisation"[All Fields] OR "randomisations"[All Fields] OR "randomise"[All Fields] OR "randomised"[All Fields] OR "randomising"[All Fields] OR "randomizations"[All Fields] OR "randomize"[All Fields] OR "randomizes"[All Fields] OR "randomizing"[All Fields] OR "randomness"[All Fields] OR "randoms"[All Fields] OR ("random allocation"[MeSH Terms] OR ("random"[All Fields] AND "allocation"[All Fields]) OR "random allocation"[All Fields] OR "randomization"[All Fields] OR "randomized"[All Fields] OR "random"[All Fields] OR "randomisation"[All Fields] OR "randomisations"[All Fields] OR "randomise"[All Fields] OR "randomised"[All Fields] OR "randomising"[All Fields] OR "randomizations"[All Fields] OR "randomize"[All Fields] OR "randomizes"[All Fields] OR "randomizing"[All Fields] OR "randomness"[All Fields] OR "randoms"[All Fields]))	138	14:50:15

Search	Actions	Details	Query	Results	Time
			**Translations**<br>**randomized:** "random allocation"[MeSH Terms] OR ("random"[All Fields] AND "allocation"[All Fields]) OR "random allocation"[All Fields] OR "randomization"[All Fields] OR "randomized"[All Fields] OR "random"[All Fields] OR "randomisation"[All Fields] OR "randomisations"[All Fields] OR "randomise"[All Fields] OR "randomised"[All Fields] OR "randomising"[All Fields] OR "randomizations"[All Fields] OR "randomize"[All Fields] OR "randomizes"[All Fields] OR "randomizing"[All Fields] OR "randomness"[All Fields] OR "randoms"[All Fields]<br>**randomised:** "random allocation"[MeSH Terms] OR ("random"[All Fields] AND "allocation"[All Fields]) OR "random allocation"[All Fields] OR "randomization"[All Fields] OR "randomized"[All Fields] OR "random"[All Fields] OR "randomisation"[All Fields] OR "randomisations"[All Fields] OR "randomise"[All Fields] OR "randomised"[All Fields] OR "randomising"[All Fields] OR "randomizations"[All Fields] OR "randomize"[All Fields] OR "randomizes"[All Fields] OR "randomizing"[All Fields] OR "randomness"[All Fields] OR "randoms"[All Fields]		

Showing 1 to 1 of 1 entries

SciELO Citation Index:

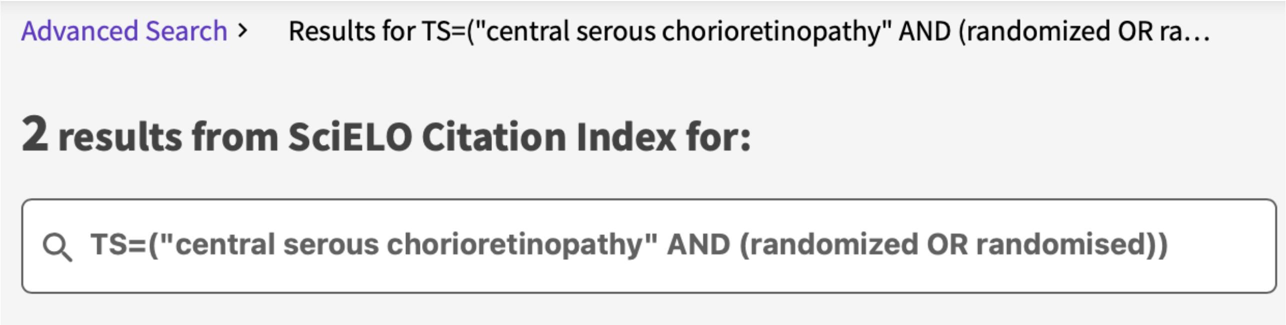

Web of Science Core Collection:

Advanced Search › Results for TS=("central serous chorioretinopathy" AND (randomized OR ra…

126 results from Web of Science Core Collection for:

TS=("central serous chorioretinopathy" AND (randomized OR randomised))
