# Supplementary material for: Scientific Literature Database Coverage of Randomized Clinical Trials for Central Serous Chorioretinopathy
Source: J Pers Med. 2023 Jun 12;13(6):983. doi: 10.3390/jpm13060983 (PMC10305376; doi:10.3390/jpm13060983)
Supplement: Supplementary file 1 [file jpm-13-00983-s001.zip › Supplementary File S2.pdf]

Supplementary File S2. Summary of randomized clinical trials identified through literature search.

Abrishami, M., et al. (2015). "Treatment of Chronic Central Serous Chorioretinopathy with Oral Methotrexate." Journal of Ocular Pharmacology and Therapeutics **31**(8): 468-475.

Purpose: To evaluate the effects of oral methotrexate (MTX) in patients with chronic central serous chorioretinopathy (CSC). Methods: This is an interventional, prospective uncontrolled clinical trial, which included 23 eyes of 23 consecutive patients presenting with chronic symptomatic CSC and persistent subretinal fluid (SRF) for longer than 3 months. All patients were treated with 7.5mg/week of oral MTX for 12 weeks. The best corrected visual acuity (BCVA), central macular thickness (CMT), SRF, and total macular volume recorded by monthly optical coherence tomography were analyzed. Complete blood count and serum liver enzymes level were monitored. Results: Mean duration of CSC was 13 months (3-36 months). Mean BCVA improved from 20/40 at baseline to 20/30 at the third month and 20/28 at the sixth month (P=0.002 and 0.003, respectively). Mean CMT decreased from 375m at baseline to 278m and 265m at the third and sixth month (P=0.002 and 0.007, respectively). Mean total macular volume decreased from 9.33mm(3) at baseline to 8.48 and 8.31mm(3) at the third and sixth month (P=0.001 and 0.001, respectively). Thirteen (62%) eyes achieved complete resolution of SRF. No MTX-associated toxicity was detected. Conclusion: Low-dose oral MTX may be an alternative therapeutic option for the treatment of chronic CSC. This study paves the way for a randomized clinical trial comparing the effects of MTX treatment with photodynamic therapy or observation.

Ambiya, V., et al. (2017). "Early focal laser photocoagulation in acute central serous chorioretinopathy: a prospective, randomized study." Ophthalmic surgery lasers and imaging retina **48**(7): 564-571.

Background and Objective: To evaluate the role of early focal laser photocoagulation in acute central serous chorioretinopathy (CSC). PATIENTS AND METHODS: A total of 58 eyes with acute naive CSC (less than 2 months' duration) with focal leak on fundus fluorescein angiography (FFA) were randomized into either a laser or a sham laser group. Eyes with chronic CSC and subfoveal leak were excluded. Visual acuity assessment, microperimetry, optical coherence tomography, and FFA were done at baseline, 1 month (minus FFA), 3 months, and 6 months after treatment. RESULTS: There was a signifcant improvement in best-corrected visual acuity, low-contrast visual acuity, retinal sensitivity, and central macular thickness at all visits in both groups (P >.001); however, there was no signifcant difference between the groups regarding time of resolution. Four eyes in the sham laser group needed rescue laser compared with one eye in early laser group (P =.16). CONCLUSION: Early laser photocoagulation is not superior to sham laser for acute CSC; therefore, observation appears to the safest and most effective strategy.

Ambiya, V., et al. (2023). "532 nm versus 810 nm subthreshold micropulse laser in treatment of non-resolving central serous chorioretinopathy: a randomized controlled trial." Armed Forces medical journal, India.

Background: The relative efficacy of 532 nm subthreshold micropulse laser in comparison to the 810 nm subthreshold micropulse laser, in the treatment of central serous chorioretinopathy is not known. Methods: This randomized controlled trial included 99 eyes each in groups A and B. Key inclusion criteria were: (i) vision loss for minimum three months due to persistent central serous chorioretinopathy; (ii) focal leaks (upto two leaks) on fundus fluorescein angiography. Key exclusion criteria were: (i) history of prior treatment for central serous chorioretinopathy; (ii) absence of any leak/ presence of diffuse leaks/ >2 leaks on fundus fluorescein angiography; (iii) chronic central serous chorioretinopathy. All eyes were treated with subthreshold micropulse laser (group A: 532 nm green laser; group B: 810 nm diode laser). Best-corrected visual acuity,

autofluorescence, spectral domain optical coherence tomography, and fundus fluorescein angiography, were evaluated at baseline and at 1, 3, and 6 months. Laser was repeated in nor-responders at 3 months. Results: There was a statistically significant improvement in BCVA in both groups six months post laser. Between the two groups, a comparable proportion of eyes showed complete resolution of subretinal fluid at one month, three months and at six months. No adverse effect of laser was observed in either group. Conclusion: Both 532 nm STMP laser and 810 nm STMP laser have comparable structural and functional outcomes in the treatment of non-resolving CSC. There are no adverse effects related to either of the two wavelengths.

Arora, S., et al. (2019). "Subthreshold diode micropulse laser versus observation in acute central serous chorioretinopathy." Clinical & experimental optometry **102**(1): 79-85.

BACKGROUND: The purpose of this study was to evaluate subthreshold diode micropulse (SDM) laser as a treatment modality in acute central serous chorioretinopathy (CSC) and compare it with the current standard of care (observation). METHODS: A randomised controlled trial was conducted on 68 eyes (34 eyes in SDM laser group and 34 eyes in observation group) with acute CSC, with a single angiographic leak and duration of complaints less than two months. Detailed history, examination and investigations were performed at the baseline and at regular intervals until six months. RESULTS: Eyes in the laser group had significantly higher best-corrected visual acuity at two weeks ($p = 0.002$), four weeks ($p < 0.001$), eight weeks ($p < 0.001$), 16 weeks ($p = 0.042$) and six months ($p = 0.008$), and higher contrast sensitivity at eight weeks ($p = 0.008$), 16 weeks ($p < 0.001$) and six months ($p < 0.001$). A recurrent/persistent neurosensory detachment was observed at the end of six months in 11.76 per cent of SDM laser treated eyes versus 29.41 per cent of eyes in the observation group ($p = 0.036$). CONCLUSION: SDM laser produces faster and superior visual rehabilitation without any adverse effects. It also reduces the chances of CSC going into chronicity and recurrence compared to the current standard of care (observation).

Bae, S. H., et al. (2014). "Low-fluence photodynamic therapy versus ranibizumab for chronic central serous chorioretinopathy: one-year results of a randomized trial." Ophthalmology **121**(2): 558-565.

PURPOSE: To compare the efficacy and safety between low-fluence photodynamic therapy (PDT) and the intravitreal ranibizumab in the treatment of chronic central serous chorioretinopathy (CSC). DESIGN: Prospective, randomized, single-center, parallel-arm, controlled trial. PARTICIPANTS: Thirty-four eyes of 32 patients with chronic CSC with >6 months' duration of symptoms or recurrent CSC were randomly placed into the low-fluence PDT group ($n = 18$) or the ranibizumab group ($n = 16$). INTERVENTION: The patients underwent a single session of low-fluence PDT or 3 consecutive monthly injections of ranibizumab. Rescue treatment was available from month 3 if the subretinal fluid (SRF) persisted or recurred after primary treatment; low-fluence PDT was given to the ranibizumab group and intravitreal ranibizumab to the low-fluence PDT group. MAIN OUTCOME MEASURES: The primary outcome was the proportion of eyes with complete resolution of SRF without rescue treatment. Secondary outcomes included the mean changes in logarithm of the minimum angle of resolution best-corrected visual acuity (BCVA), central retinal thickness (CRT), and angiographic findings from baseline to 12 months. RESULTS: At month 12, 16 eyes (88.9%) of the low-fluence PDT group maintained complete resolution of SRF without rescue treatment versus 2 eyes (12.5%) in the ranibizumab group ($P <0.001$). Two eyes (11.1%) in the low-fluence PDT group and 11 eyes (68.8%) in the ranibizumab group met the criteria for rescue treatment ($P = 0.001$). In the low-fluence PDT group, the mean decrease in CRT from baseline was significantly greater than that in the ranibizumab group until month 6 ($P <0.05$), but the differences became insignificant thereafter. The improvement in BCVA from baseline was superior in the low-fluence PDT group to that in the ranibizumab group, but the differences were

not statistically significant except at month 3 (P = 0.025). On indocyanine green angiography, a significantly greater proportion of the low-fluence PDT group (16 eyes; 88.9%) showed a marked reduction in choroidal hyperpermeability after primary treatment than that of the ranibizumab group (0 eyes; P <0.001). No serious adverse events related to the drugs or procedures were observed. CONCLUSIONS: This study represents the overall superiority of low-fluence PDT compared with intravitreal ranibizumab in the treatment of chronic CSC.

Bae, S. H., et al. (2011). "A randomized pilot study of low-fluence photodynamic therapy versus intravitreal ranibizumab for chronic central serous chorioretinopathy." American journal of ophthalmology **152**(5): 784-792.e782.

Purpose: To report 6-month outcomes of a prospective, randomized study comparing the efficacy and safety between low-fluence photodynamic therapy (PDT) and intravitreal injections of ranibizumab in the treatment of chronic central serous chorioretinopathy.Design: Prospective, randomized, single-center pilot study.Methods: Sixteen eyes with chronic central serous chorioretinopathy were randomized to receive either low-fluence PDT or intravitreal injections of ranibizumab: 8 eyes in the low-fluence PDT group and 8 in the ranibizumab group. Rescue treatment was considered if subretinal fluid was sustained after completion of primary treatment: low-fluence PDT for the ranibizumab group and ranibizumab injection for the low-fluence PDT group. Main outcome measures were excess foveal thickness, resolution of subretinal fluid, choroidal perfusion on indocyanine green angiography, and best-corrected visual acuity.Results: At 3 months, the mean excess foveal thickness was reduced from 74.1 ± 56.0 μm to -35.4 ± 44.5 μm in the low-fluence PDT group (P = .017) and from 26.3 ± 50.6 μm to -23.1 ± 56.5 μm in the ranibizumab group (P = .058). After a single session of PDT, 6 eyes (75%) in the low-fluence PDT group achieved complete resolution of subretinal fluid and reduction of choroidal hyperpermeability, whereas 2 (25%) eyes in the ranibizumab group achieved this after consecutive ranibizumab injections. Four eyes (50%) in the ranibizumab group underwent additional low-fluence PDT and accomplished complete resolution. At 3 months, significant improvement of best-corrected visual acuity was not demonstrated in the low-fluence PDT group (P = .075), whereas it was observed in the ranibizumab group (P = .012). However, the tendency toward improvement of best-corrected visual acuity was not maintained.Conclusions: In terms of anatomic outcomes, the effect of ranibizumab injections was not promising compared with that of low-fluence PDT.

Behnia, M., et al. (2013). "Improvement in visual acuity and contrast sensitivity in patients with central serous chorioretinopathy after macular subthreshold laser therapy." Retina (Philadelphia, Pa.) **33**(2): 324-328.

PURPOSE: The purpose of the study was to measure the improvement in vision and contrast sensitivity in patients with central serous chorioretinopathy after macular subthreshold laser therapy. METHODS: Cases included in this clinical trial were patients diagnosed with acute central serous chorioretinopathy less than 1 month in duration. Each patient was randomized to either observation group or treatment group with subthreshold argon laser. First and sixth month of follow-up results were then recorded, and visual improvement and contrast sensitivity were compared between the two groups. RESULTS: Of 37 patients with central serous chorioretinopathy, 25 were men and 12 were women with a mean age of 38.6 ± 5.9 years. At 6 months, mean best-corrected visual acuity improved from 0.26 to 0.03 logarithm of the minimum angle of resolution in the laser group and from 0.12 to 0.04 logarithm of the minimum angle of resolution in the observation group (P < 0.001). Except spatial frequencies of 18 cycles per degree (P = 0.207), the contrast sensitivity in spatial frequencies of 3, 6, and 12 cycles per degree was significantly more favorable in the laser group during the first and sixth month. CONCLUSION:

Treatment of patients with acute central serous chorioretinopathy with subthreshold laser improved the best-corrected visual acuity and contrast sensitivity compared with those in the observation group. The laser group also showed a more rapid improvement. In both groups, best-corrected visual acuity improved in most patients after 6 months; however, the contrast sensitivity was significantly better in the treatment group.

Boscia, F., et al. (2008). "Low fluence photodynamic therapy in chronic central serous chorioretinopathy: blind randomised clinical trial of efficacy and safety." Investigative ophthalmology & visual science: ARVO E-abstract 3278.

Purpose: : To evaluate in a blind randomized clinical trial the efficacy and safety profile of photodinamic therapy (PDT) with a low fluence rate for treatment of chronic central serous chorioretinopathy (CSC) compared to an untrated group. Methods: : Sixteen eyes with chronic CSC were randomized into 2 groups to receive either PDT with verteporfin (fluence 25 J/cm2, light dose rate: 300 mW/cm2) or no therapy. Inclusion criteria were: Best-corrected visual acuity (BCVA) between 0,2 and 1 logMAR; presence of subretinal fluid and/or serous pigment epithelial detachment on optical coherence tomography (OCT) without regression for 3 or more months, RPE leakage on fluorescein angiography and choroidal vascular hyperpermeability on confocal scanning laser indocyanine green angiography (SLO-ICGA). Exclusion criteria were: any previous treatment for CSC; evidence of other chorioretinal disorders; media opacities; and treatment with systemic steroids. The laser irradiation was applied on the areas of choroidal vascular hyperpermeability as observed on ICGA. Nonconfluent laser spots were used in case of multiple hyperpermeable areas, including the fovea, if involved. Primary outcome measures were: far BCVA (logMAR, using ETDRS charts) and near BCVA (logMAR, using MNRead Acuity Charts), central macular thickness (OCT3, Zeiss-Humphrey); secondary outcome measures were macular sensitivity and stability of fixation determined using microperimetry (Nidek MP1). All outcome measures were evaluated 1, 4, 12 and 24 weeks after treatment. Results: : No significant changes in all parameters were seen in control untreated group. A significant improvement of far and near BCVA were seen in comparison with both baseline (ANOVA, p=0,008 and 0,000), and the control group (t-TEST, p=0,010 and p=0,000), with the greater effect on week 24. In all eyes treated with low fluence PDT a complete resolution of subretinal fluid was observed, with a significant reduction of central macular thickness. On week 24, in treated eyes a significant improvement in mean fixation stability was also observed (ANOVA, p=0,011). No recurrence was seen during the follow-up period. No adverse event, like persistent occlusive effect on the choriocapillaris, pigmentary changes in the treatment zone, or choroidal neovascularization occurred in any of the treated patients. Conclusions: : Low fluence PDT is effective and safe for treating chronic CSC.

Bousquet, E., et al. (2015). "Spironolactone for nonresolving central serous chorioretinopathy a randomized controlled crossover study." Retina **35(12)**: 2505-2515.

Purpose: To evaluate the effect of spironolactone, a mineralocorticoid receptor antagonist, for nonresolving central serous chorioretinopathy. Method(s): This is a prospective, randomized, double-blinded, placebo-controlled crossover study. Sixteen eyes of 16 patients with central serous chorioretinopathy and persistent subretinal fluid (SRF) for at least 3 months were enrolled. Patients were randomized to receive either spironolactone 50 mg or placebo once a day for 30 days, followed by a washout period of 1 week and then crossed over to either placebo or spironolactone for another 30 days. The primary outcome measure was the changes from baseline in SRF thickness at the apex of the serous retinal detachment. Secondary outcomes included subfoveal choroidal thickness and the ETDRS best-corrected visual acuity. Result(s): The mean duration of central serous chorioretinopathy before enrollment in study eyes was 10 +/- 16.9 months. Crossover data

analysis showed a statistically significant reduction in SRF in spironolactone treated eyes as compared with the same eyes under placebo (P = 0.04). Secondary analysis on the first period (Day 0-Day 30) showed a significant reduction in subfoveal choroidal thickness in treated eyes as compared with placebo (P = 0.02). No significant changes were observed in the best-corrected visual acuity. There were no complications related to treatment observed. Conclusion(s): In eyes with persistent SRF due to central serous chorioretinopathy, spironolactone significantly reduced both the SRF and the subfoveal choroidal thickness as compared with placebo.

Bousquet, E., et al. (2013). "Mineralocorticoid receptor antagonism in the treatment of chronic central serous chorioretinopathy: A pilot study." Retina **33(10)**: 2096-2102.

PURPOSE: Based on experimental data showing that central serous chorioretinopathy could result from overactivation of mineralocorticoid receptor pathway in choroid vessels, the authors studied eplerenone, a mineralocorticoid receptor antagonist, as a potential treatment for chronic central serous chorioretinopathy. METHOD(S): This nonrandomized pilot study included 13 patients with central serous chorioretinopathy of at least 4-month duration, treated with 25 mg/day of oral eplerenone for a week followed by 50 mg/day for 1 or 3 months. The primary outcome measure was the changes in central macular thickness recorded by optical coherence tomography, and the secondary outcomes included changes in foveal subretinal fluid (SRF) measured by OCT, in best-corrected visual acuity (BCVA) and the percentage of eyes achieving complete resolution of subretinal fluid during the treatment period. RESULT(S): Central macular thickness decreased significantly from 352 +/- 139 mum at baseline to 246 +/- 113 mum and 189 +/- 99 mum at 1 and 3 months under eplerenone treatment (P < 0.05 and P < 0.01, respectively). At 3 months, the subretinal fluid significantly decreased compared with baseline subretinal fluid (P < 0.01) and best-corrected visual acuity significantly improved compared with baseline best-corrected visual acuity (P < 0.001). CONCLUSION(S): Eplerenone treatment was associated with a significant reduction in central macular thickness, subretinal fluid level, and an improvement in visual acuity. Randomized controlled trials are needed to confirm these encouraging results.

Chan, P. S. T., et al. (2006). "Low-Dose Transpupillary Thermotherapy for the Treatment of Central Serous Chorioretinopathy." American academy of ophthalmology: 290.

Purpose: To determine the safety and efficacy of low-dose transpupillary thermotherapy (TTT) for the treatment of central serous chorioretinopathy (CSR). Methods: Fifteen eyes of 15 patients with CSR were randomly assigned to receive low-dose TTT (100 mW/mm2) or sham therapy (control). The follow-up period was three months. Results: CSR resolved in 8 of 8 (100%) treated eyes compared to 5 of 7 (71 %) control eyes (P= .05). There were no adverse events resulting from TTT. Conclusion: Low-dose TTT is a promising treatment for CSR and may effect faster disease resolution.

Chan, W. M., et al. (2008). "Half-Dose Verteporfin Photodynamic Therapy for Acute Central Serous Chorioretinopathy. One-Year Results of a Randomized Controlled Trial." Ophthalmology **115(10)**: 1756-1765.

Objective: To evaluate the efficacy of photodynamic therapy (PDT) with half-dose verteporfin for treating acute central serous chorioretinopathy (CSC). Design(s): Prospective, double-masked, placebo-controlled, randomized clinical trial. Participants and Controls: Sixty-three eyes of 63 patients with acute symptomatic CSC of 3 months' duration or less were recruited. Forty-three eyes were randomized to indocyanine green angiography (ICGA)-guided PDT with half-dose (3 mg/m²) verteporfin and 21 eyes were randomized to placebo. Intervention(s): Patients in the verteporfin group received an infusion of half-dose verteporfin over 8 minutes, followed by

ICGA-guided PDT 10 minutes from the start of infusion. Laser was applied for 83 seconds covering the choroidal abnormalities observed in ICGA, with a maximum laser spot size of 4500 mum. Main Outcome Measure(s): The primary outcome measure was the proportion of eyes with absence of subretinal fluid at the macula at 12 months. Secondary outcome measures included changes in mean logarithm of the minimum angle of resolution (logMAR) best-corrected visual acuity (BCVA), subjective symptoms, optical coherence tomography (OCT) results, central foveal thickness (CFT), and angiographic findings during the 12-month study period. Result(s): Thirty-nine patients in the verteporfin group and 19 patients in the placebo group completed 12 months of follow-up. Thirty-seven (94.9%) eyes in the verteporfin group compared with 11 (57.9%) eyes in the placebo group showed absence of subretinal fluid at the macula at 12 months (P = 0.001). The mean logMAR BCVA at 12 months was significantly better in the verteporfin group compared with the placebo group: -0.05 and 0.05, respectively (P = 0.008). All 39 (100%) verteporfin-treated eyes had stable or improved vision, compared with 15 (78.9%) eyes in the placebo group (P = 0.009). The mean OCT CFT for the verteporfin group also was significantly lower compared with the placebo group at 12 months (P = 0.001). No ocular or systemic adverse event was encountered in the study. Conclusion(s): Photodynamic therapy with half-dose verteporfin is effective in treating acute symptomatic CSC, resulting in a higher proportion of patients with absence of exudative macular detachment and better visual acuity compared with placebo. Financial Disclosures(s): Proprietary or commercial disclosure may be found after the references. © 2008 American Academy of Ophthalmology.

Chen, L. C., et al. (2020). "Oral treatment of central serous chorioretinopathy patients using propranolol tablets." Pharmaceuticals (Basel, Switzerland) **13**(11): 1-14.

Purpose: To evaluate the pharmacological effects of propranolol treatment of patients with central serous chorioretinopathy (CSCR) over 4 months. Results: Among the 89 male and 31 female patients, the mean BCVA decreased to 0.42 ± 0.08 logMAR during CSCR attacks. Oral propranolol showed good effectiveness in reducing CSCR signs after at least 4 months of treatment. The final BCVA of the patients in groups 1 and 2 was 0.09 ± 0.01 and 0.19 ± 0.03 logMAR, respectively ($p < 0.05$). Moreover, the mean complete remission time in groups 1 and 2 was 1.9 and 3.5 months, respectively ($p < 0.05$), while the “success” rate in groups 1 and 2 was 95.0% (57/60) and 78.3% (47/60), respectively ($p <0.05$). The recurrence rate in groups 1 and 2 was 5.3% (3/57) and 25.5% (12/47) after a further 5 months of follow-up, respectively ($p < 0.05$). Materials and Methods: One hundred and twenty patients were enrolled and randomly divided into two groups that both underwent a visual acuity test and optical coherence tomography (OCT) scanning, between April and December 2017. The 60 patients in group 1 were requested to take propranolol for 4 months, while the other 60 subjects (group 2) received placebo therapy during the same period. The best-corrected visual acuity (BCVA) of every volunteer and an OCT image of each patient were checked and recorded at the beginning of the study and each week thereafter. If the signs of CSCR disappeared completely from the OCT scans, the case was considered a “success” and treatment stopped at once. However, the “success” subjects were further evaluated in follow-ups throughout the next 5 months to determine the rate of recurrence in groups 1 and 2. The time of total complete remission of CSCR from the OCT scans was also measured in groups 1 and 2. Conclusion: CSCR patients revealed an excellent prognosis and success rate of 95.0% after taking propranolol. The treatment was able to enhance subretinal fluid (SRF) absorption, shorten the time to total complete remission, and significantly decrease CSCR recurrence. As such, we suggest that taking propranolol may be an alternative and viable choice for CSCR patients, given that the new method was shown to be safe, cheap, effective, well tolerated and convenient.

Cheng, C.-K., et al. (2017). "COMPARISON OF PHOTODYNAMIC THERAPY USING HALF-DOSE OF VERTEPORFIN OR HALF-FLUENCE OF LASER LIGHT FOR THE TREATMENT OF CHRONIC CENTRAL SEROUS CHORIORETINOPATHY." Retina **37**(2): 325-333.

Purpose: To compare the efficacy and the detrimental effects of half-drug dose and half-laser light fluence of photodynamic therapy (PDT) for the treatment of chronic central serous chorioretinopathy.Design: We conducted a prospective randomized, observer-masked comparison study.Methods: Forty eyes (40 patients) with chronic central serous chorioretinopathy were enrolled in this study and were equally divided into 2 groups. The first (half-dose) group received only half the standard dose of verteporfin infusion (3 mg/m(2)) and were irradiated by the standard 83 seconds of laser light (50 J/cm(2)) for the PDT treatment; the second (half-fluence) group received the standard dose of verteporfin infusion (6 mg/m2) and were irradiated by only 42 seconds of laser light (25 J/cm(2)). Patients were examined at baseline and 1 week, 1 month, 3 months, and 6 months after PDT treatments with best-corrected visual acuity and optical coherence tomography. Fluorescein angiography and indocyanine green angiography (ICGA) were performed at baseline and at 1 month, 3 months, and 6 months after PDT treatment. Primary outcome measures were the changes in the best-corrected visual acuity and in central retinal thickness and subretinal fluid in optical coherence tomography. Secondary outcomes were the changes in the choroidal perfusion in the ICGA, which was measured as the fluorescein ratio of the PDT-treated area to a nontreated reference area in ICGA.Results: Best-corrected visual acuity was significantly improved at post-PDT 1 month, 3 months, and 6 months (all $P < 0.01$) in both the half-dose and the half-fluence group. Central retinal thickness was significantly improved at all post-PDT time points in both groups ($P < 0.05$). All patients in the half-dose group and 19 patients (95%) in the half-fluence group had complete absorption of subretinal fluid at post-PDT 3 months and 6 months. The choroidal perfusion (as reflected by the decrease of the ratio of fluorescence) in ICGA was significantly decreased at all post-PDT follow-up time points in both groups ($P < 0.01$). However, there were no significant differences in all the measurements between the two groups, including best-corrected visual acuity, central retinal thickness, and hypo-fluorescence in ICGA at baseline and at each post-PDT follow-up time point.Conclusion: Both half-dose and half-fluence modifications of PDT were similarly effective in improving the visual acuity and subretinal fluid for chronic CSC. Both types of modification of PDT were also similar in causing postlaser choroidal hypoperfusion.

Dang, Y., et al. (2013). "The effect of eradicating Helicobacter pylori on idiopathic central serous chorioretinopathy patients." Therapeutics and clinical risk management **9**(1): 355-360.

Purpose: To evaluate the effect of Helicobacter pylori (H. pylori) eradication on the remission of acute idiopathic central serous chorioretinopathy (ICSCR). Study design: A prospective, randomized, placebo-controlled study of 53 participants. Main outcome measure: Twenty-seven acute ICSCR patients tested positive for H. pylori were given an eradication H. pylori therapy, and another 26 patients with the same diagnosis received matching placebo medication. All participants were tested for the following items: (1) disappearance rate of subretinal fluid (SRF); (2) best-corrected visual acuity (BCVA); and (3) central retinal sensitivity at baseline, 2 weeks, 4 weeks, 8 weeks, and 12 weeks after treatment. The difference between the two groups was analyzed by PASW statistics version 18.0. Results: At each follow-up, the disappearance rate of SRF in the active treatment group seemed slightly better than in the control group, but no statistically significant differences were observed ($P > 0.05$ at each follow-up). The BCVA between the two groups also did not demonstrate statistically significant differences ($P > 0.05$ at each follow-up). Unlike the BCVA and the disappearance rate of SRF, we compared the change in central retinal sensitivity at 12 weeks after treatment; a statistical difference was observed ($P = 0.042$).

Conclusion: Our findings suggested that H. pylori eradication does not improve BCVA and the disappearance rate of SRF, but it could improve the central retinal sensitivity in acute ICSCR patients. We recommend that chronic ICSCR patients and more sensitive methods for H. pylori diagnosis should be involved in evaluating the effect of H. pylori eradication. © 2013 Dang et al.

de la Cruz-Merino, L., et al. (2017). "Clinical features of serous retinopathy observed with cobimetinib in patients with BRAF-mutated melanoma treated in the randomized coBRIM study." J Transl Med **15**(1): 146.

BACKGROUND: Serous chorioretinopathy has been associated with MEK inhibitors, including cobimetinib. We describe the clinical features of serous retinopathy observed with cobimetinib in patients with BRAF (V600)-mutated melanoma treated in the Phase III coBRIM study. METHODS: In the coBRIM study, 493 patients were treated in two randomly assigned treatment groups: cobimetinib and vemurafenib (n = 247) or vemurafenib (n = 246). All patients underwent prospective ophthalmic examinations at screening, at regular intervals during the study, and whenever ocular symptoms developed. Patients with serous retinopathy were identified in the study database using a group of relevant and synonymous adverse event terms. RESULTS: Eighty-six serous retinopathy events were reported in 70 patients (79 events in 63 cobimetinib and vemurafenib-treated patients vs seven events in seven vemurafenib-treated patients). Most patients with serous retinopathy identified by ophthalmic examination had no symptoms or had mild symptoms, among them reduced visual acuity, blurred vision, dyschromatopsia, and photophobia. Serous retinopathy usually occurred early during cobimetinib and vemurafenib treatment; median time to onset was 1.0 month. Most events were managed by observation and continuation of cobimetinib without dose modification and resolved or were resolving by the data cutoff date (19 Sept 2014). CONCLUSIONS: Cobimetinib treatment was associated with serous retinopathy in patients with BRAF (V600)-mutated melanoma. Retinopathy was generally asymptomatic or mild. Periodic ophthalmologic evaluations at regular intervals and at the manifestation of any visual disturbance are recommended to facilitate early detection and resolution of serous retinopathy while patients are taking cobimetinib. Trial Registration Clinicaltrials.gov (NCT01689519). First received: September 18, 2012.

Feenstra, H. M. A., et al. (2023). "Increasing Evidence for the Safety of Fovea-Involving Half-Dose Photodynamic Therapy for Chronic Central Serous Chorioretinopathy." Retina **43(3)**: 379-388.

Purpose:A retrospective study was performed with data from the prospective randomized controlled trials, PLACE and SPECTRA, assessing the risk of foveal atrophy and the likelihood of structural and functional improvement on optical coherence tomography, after foveal half-dose photodynamic therapy in chronic central serous chorioretinopathy. Method(s):A total of 57 chronic central serous chorioretinopathy patients received a single half-dose photodynamic therapy with a treatment spot that included the fovea. Optical coherence tomography scans and fundus autofluorescence images were analyzed for structural improvement and possible atrophy development, at baseline and at several visits after treatment. Main outcome measures were integrity of the external limiting membrane and ellipsoid zone on optical coherence tomography and hypoautofluorescence on fundus autofluorescence. Result(s):The subfoveal external limiting membrane was graded as continuous in 21 of 57 of patients (36.8%) at baseline, and the subfoveal ellipsoid zone was graded as continuous in 5 of 57 patients (8.8%) at first visit, which improved to 50 of 51 (98.0%) and 32 out of 51 (62.7%) at the final visit at 2 years, respectively (both $P < 0.001$). Hypoautofluorescent changes on fundus autofluorescence were present in 25 of 55 patients (45.5%) at baseline and in 23 of 51 patients (45.1%) at the final visit ($P = 0.480$). Conclusion(s):In patients with chronic central serous chorioretinopathy who received a single, foveal, half-dose photodynamic therapy, a

significant improvement in structure and function was seen at the final follow-up. None of the patients developed foveal atrophy. Copyright © 2022 Wolters Kluwer Health, Inc. All rights reserved.

Feenstra, H. M. A., et al. (2022). "EFFICACY OF HALF-DOSE PHOTODYNAMIC THERAPY VERSUS HIGH-DENSITY SUBTHRESHOLD MICROPULSE LASER FOR TREATING PIGMENT EPITHELIAL DETACHMENTS IN CHRONIC CENTRAL SEROUS CHORIORETINOPATHY." Retina (Philadelphia, Pa.) **42**(4): 721-729.

PURPOSE: Comparing the effect of half-dose photodynamic therapy and high-density subthreshold micropulse laser treatment on retinal pigment epithelial detachments (PEDs) in chronic central serous chorioretinopathy. METHODS: This study included data from the PLACE trial, a prospective randomized controlled trial comparing half-dose photodynamic therapy and high-density subthreshold micropulse laser treatment in chronic central serous chorioretinopathy. Main outcome measurements were changes in both the foveal PED and the highest PED within the macula at baseline compared with first and final evaluation visit. RESULTS: At baseline, a macular PED was detected in 76.9% of patients (123/160), and a PED within 1,500 μm from the foveal center in 37.5% of patients (60/160). In the half-dose photodynamic therapy arm (61 patients), there was a significantly larger decrease in the highest macular PED compared with the high-density subthreshold micropulse laser treatment arm (62 patients) at both first and final evaluation visits ($P < 0.001$ and $P = 0.012$, respectively). The decrease of highest foveal PED was significant at first visit ($P = 0.025$). CONCLUSION: Half-dose photodynamic therapy is superior to high-density subthreshold micropulse laser treatment with regard to a statistically significant reduction in the height of macular PEDs in active chronic central serous chorioretinopathy. These findings may also have implications for other diseases within the pachychoroid disease spectrum that can present with PEDs.

Feenstra, H. M. A., et al. (2023). "Long-term follow-up of chronic central serous chorioretinopathy patients after primary treatment of oral eplerenone or half-dose photodynamic therapy and crossover treatment: SPECTRA trial report No. 3." Albrecht von Graefes Archiv fur klinische und experimentelle Ophthalmologie [Graefe's archive for clinical and experimental ophthalmology] **261**(3): 659-668.

Purpose: Comparing anatomic and functional efficacy and safety of primary treatment with either half-dose photodynamic therapy (PDT) or oral eplerenone, or crossover treatment in chronic central serous chorioretinopathy patients. Methods: After the SPECTRA trial baseline visit, patients were randomized to either half-dose PDT or eplerenone and received crossover treatment if persistent subretinal fluid (SRF) on optical coherence tomography (OCT) was present at first follow-up (at 3 months). Presence of SRF and best-corrected visual acuity (BCVA) was evaluated at 12 months. Results: Out of the 90 patients evaluated at 12 months, complete SRF resolution was present on OCT in 43/48 (89.6%) of patients who were primarily randomized to half-dose PDT and in 37/42 (88.1%) who were primarily randomized to eplerenone. Out of the 42 patients that were primarily randomized to eplerenone, 35 received crossover treatment with half-dose PDT. The BCVA improved significantly more at 12 months in patients who had received primary half-dose PDT as compared to the primary eplerenone group ($p = 0.030$). Conclusions: Twelve months after baseline visit, most patients treated with half-dose PDT (either primary or crossover treatment) still had complete SRF resolution. The long-term BCVA in patients who receive primary half-dose PDT is better than in patients in whom PDT is delayed due to initial eplerenone treatment with persistent SRF.

Feenstra, H. M. A., et al. (2022). "Crossover to Half-dose Photodynamic Therapy or Eplerenone in Chronic Central Serous Chorioretinopathy patients (SPECS)." Ophthalmology. retina.

PURPOSE: Comparing the efficacy and safety of crossover treatment to half-dose photodynamic therapy (PDT) and eplerenone treatment after failure of primary treatment in chronic central serous chorioretinopathy (cCSC) patients. DESIGN: Multicenter crossover clinical trial. SUBJECTS: At 3 months after baseline visit of the SPECTRA randomized controlled trial, either half-dose PDT or eplerenone treatment was evaluated, and patients who still demonstrated subretinal fluid (SRF) were included in the current study, the SPECS trial. METHODS: At the baseline visit of the current SPECS trial, crossover treatment was performed in patients who still demonstrated subretinal fluid (SRF). These subjects received either half-dose PDT or oral eplerenone for 12 weeks. Both anatomical and functional parameters were evaluated 3 months after crossover treatment. RESULTS: Forty-nine patients were included in the SPECS trial (38 received primary eplerenone treatment and 11 half-dose PDT). At 3 months after crossover treatment, 32/37 (86.5%) in the crossover to half-dose PDT group and 2/9 (22.2%) in the crossover to eplerenone group had complete SRF resolution (p=0.030). The mean foveal sensitivity increased significantly more in the crossover to half-dose PDT group (mean +3.08 dB) compared to the crossover to eplerenone group (mean -0.27 dB; p=0.009). CONCLUSIONS: CCSC patients with persistence of SRF after primary eplerenone treatment can benefit from half-dose PDT, which can induce a relatively fast and complete SRF resolution, together with an improvement in foveal sensitivity.

Goel, N., et al. (2021). "Multifocal electroretinography-assisted anatomical and functional evaluation of subthreshold green laser in acute central serous chorioretinopathy." Indian journal of ophthalmology **69**(9): 2341-2346.

Purpose: To compare observation versus subthreshold green laser (STL) in acute central serous chorioretinopathy (CSC) in terms of anatomical and functional outcomes. Methods: Prospective randomized interventional study. 30 eyes with the first episode of acute CSC underwent complete ophthalmologic examination, measurement of best-corrected Snellen visual acuity (BCVA), contrast sensitivity (CS), fundus fluorescein angiography (FFA), spectral-domain optical coherence tomography (SD-OCT), and multifocal electroretinography (mfERG) at baseline. Patients were randomized equally to group A (observation) or group B (STL using 532 nm wavelength applied to the leakage point). Outcome measures included BCVA, CS, central foveal thickness (CFT), and mean macular thickness (MMT) on SD-OCT and P1 amplitude and implicit time (IT) on mfERG. Patients were followed up for 6 months. Results: Mean BCVA was comparable between the two groups on follow up; however, mean CS was significantly higher in group B at 6 months (P = 0.032). CFT was significantly lower in group B at 1 month (P = 0.001) and 3 months (P = 0.049); however, this difference was not maintained at 6 months (P = 0.265). P1 amplitude and IT in all 5 rings were comparable between the two groups at baseline. On follow up, P1 amplitude of ring 1 became significantly higher in group B at 3 months (P = 0.036) and 6 months (P = 0.022). Conclusion: Immediate treatment of acute CSC with STL, as compared to conservative management, leads to more rapid resolution on SD-OCT and superior functional outcomes as evidenced by CS and mfERG.

Herold, T. R., et al. (2017). "Long-term results and recurrence rates after spironolactone treatment in non-resolving central serous chorio-retinopathy (CSCR)." Graefe's archive for clinical and experimental ophthalmology **255**(2): 221-229.

To evaluate the long-term results of spironolactone in non-resolving central serous chorio-retinopathy (CSCR) and recurrence rates of CSCR.Interventional uncontrolled open-label prospective clinical trial of patients with non-resolving CSCR who were treated with spironolactone

50 mg daily (Spironolacton ALA (R) 50 mg, ALIUD PHARMA) for up to 16 weeks. Follow-up visits were performed at 3, 6, 9, and 12 months. Retreatment criteria for recurrence were: gain in sub-retinal fluid (SRF) of more than 25 % plus/or increase of central retinal thickness (CRT) of more than 50 mu m plus visual symptoms compared to last visit. Main outcome measures: 12-month efficacy of upload treatment with spironolactone. Secondary outcome measure was the recurrence rate at 6, 9, and 12 months.Of the 21 study eyes treated, 71 % (n = 15) showed significant improvement or complete regression on OCT examination over 12 months. Nineteen percent of the patients (n = 4) showed a stable course from visit 1 to visit 12. The overall reduction of sub-retinal fluid from visit 1 (156 mu m +/- 131 SD) to visit 12 (53 mu m +/- 93 SD) was statistically significant (p = 0.003). The change of mean visual acuity (log MAR) from 0.25 (+/- 0.17 SD) at baseline to 0.17 (+/- 0.18 SD) at visit 12 was statistically significant, with p = 0.044.Our results confirm a positive effect of spironolactone in non-resolving CSCR in 71 % of cases. Evaluation of recurrence rates and retreatments showed good results in patients who responded to spironolactone primarily.A prospective randomized trial may provide better data about this non-invasive treatment.

Ho, M., et al. (2020). "Analysis of choriocapillaris perfusion and choroidal layer changes in patients with chronic central serous chorioretinopathy randomised to micropulse laser or photodynamic therapy." The British journal of ophthalmology. **12**.

PURPOSE: The purpose of this study was to investigate the signal changes in choriocapillaris flow deficits and choroidal thickness changes using swept-source optical coherence tomography angiography (OCTA) following different treatments. DESIGN: A double-blind, randomised controlled trial. METHOD(S): Patients with unilateral chronic central serous chorioretinopathy (CSC) were randomised to receive subthreshold micropulse laser therapy (MLT) or half-dose photodynamic therapy (PDT). Choroidal thickness and choriocapillaris flow deficit signals were investigated. RESULT(S): Eighteen patients were randomised into the MLT group and 15 patients into the PDT group. Areas with flow deficit signals were identified in all baseline OCTA images of the choriocapillaris, with mean areas of 0.420 and 0.465mm2 in the MLT and PDT groups, respectively. These flow deficit signal areas were significantly reduced at 6 months (p=0.011) in the MLT group and at 3 months (p=0.008) in the PDT group. Patients from the PDT group were shown to have smaller flow deficit areas than patients from the MLT group at all time points after treatment (p=0.001, analyses of variance). The mean choroidal volume of the fovea showed a significant reduction at 1 month (p=0.003), 3 months (p=0.199) and 6months (p=0.006) in the PDT group. CONCLUSION(S): The flow deficit areas identified in the choriocapillaris layer may suggest possible relative choroidal ischaemia. With measurement of choroidal volume reduction and faster rates of flow deficit area change, PDT has a stronger effect than MLT in promoting choriocapillaris recovery. Copyright © Author(s) (or their employer(s)) 2020. No commercial re-use. See rights and permissions. Published by BMJ.

Hu, J., et al. (2021). "OPTICAL COHERENCE TOMOGRAPHY ANGIOGRAPHY-GUIDED PHOTODYNAMIC THERAPY FOR ACUTE CENTRAL SEROUS CHORIORETINOPATHY." Retina-the Journal of Retinal and Vitreous Diseases **41**(1): 189-198.

Purpose: To compare the anatomic results of optical coherence tomography angiography (OCTA)-guided half-dose photodynamic therapy (PDT) versus indocyanine green angiography (ICGA)-guided PDT in eyes with acute central serous chorioretinopathy. Methods: This study is a prospective, single-center, noninferiority, double-masked, randomized, controlled clinical trial. Fifty-one eyes of 45 patients with acute central serous chorioretinopathy were recruited, and randomized to an ICGA-guided group and an OCTA-guided group. The primary outcome measures

were the rates of complete subretinal fluid (SRF) resolution at 1 month and 3 months. Results: Forty-six eyes of 40 patients finished the follow-up and were analyzed. In the OCTA-guided group, the SRF was completely resolved in 13 (56.5%) eyes within 1 month and in 21 (91.3%) eyes within 3 months. In the ICGA-guided group, the SRF was resolved in 16 (69.6%) of the eyes within 1 month and in 22 (95.7%) of the eyes by 3 months. Optical coherence tomography angiography-guided PDT was demonstrated noninferior to ICGA-guided PDT for SRF resolution rate at 3 months (P = 0.016), but not at 1 month (P = 0.311) for acute central serous chorioretinopathy patients. Subretinal fluid did not recur in any of the eyes in the OCTA-guided group, but did recur in 2 eyes (8.7%) of the ICGA-guided group during the 3-month follow-up period. Conclusion: Optical coherence tomography angiography-guided PDT seemed to be noninferior to ICGA-guided PDT for resolution of SRF at 3 months in eyes with acute central serous chorioretinopathy.

Jeong, K. D., et al. (2016). "Relationship between Pain and Injection Site during Intravitreal Injection." J korean ophthalmol soc **57**(6): 930-934.

PURPOSE: Using a visual analogue scale, patients pain was compared according to injection site during intravitreal injection. METHODS: A prospective, clinical trial was conducted on 171 eyes of patients experiencing age-related macular degeneration, diabetic retinopathy, retinal vein occlusion, or central serous chorioretinopathy. After determining the anatomic quadrant of the injection site, patients were randomized to receive intravitreal bevacizumab, aflibercept, ranibizumab, or dexamethasone injection. Fifteen minutes after the injection, patients completed a survey about pain using a visual analogue scale from 0 (no pain) to 10 (unbearable pain). RESULTS: According to the visual analogue scale, pain score was 3.20 at the superotemporal site, 3.03 at the superonasal site, and 2.35 at the inferonasal site. Pain was lowest when injected in an inferotemporal site (p = 0.012). CONCLUSIONS: Intravitreal injection at an inferotemporal site can help reduce patient pain.

Kapoor, K. G., et al. (2017). "Mineralocorticoid Antagonists as Adjuncts in Neovascular Age-Related Macular Degeneration." Ophthalmology and Therapy **6**(1): 141-146.

Introduction: The purpose of this project was to evaluate the role of MR antagonists as an adjunct in patients with neovascular age-related macular degeneration (AMD) who have chronic subretinal fluid. Methods: Inclusion criteria were patients with a diagnosis of neovascular AMD, who had completed at least six anti-VEGF injections, and had persistent subretinal fluid (SRF) on optical coherence tomography (OCT). Treatment with oral eplerenone was initiated and dose titrated according to protocol. Results: 23 patients were included in the study (mean age = 54.6, 52.2% female, 47.8% male). 13 of the 23 patients had predominantly chronic subretinal fluid without large PEDs. In this subgroup, mean initial central macular thickness (CMT) prior to starting oral eplerenone was 305.3 mu m, and mean injection interval was 40.25 days. Mean final CMT after at least 3 months of adjunctive eplerenone treatment was 240.6 mu m and mean injection interval with adjunctive treatment was 54.61 days. Mean extension of the injection interval after commencing oral eplerenone was 14.36 days. Conclusions: These findings suggest oral MR antagonists may have a role as an adjunctive treatment in neovascular AMD, and may be particularly useful in dehydration of the subretinal space in the setting of chronic subretinal fluid. Further research is needed in randomized controlled trials to elucidate the precise role of oral MR antagonists in neovascular AMD.

Khosla, P. K., et al. (1997). "Evaluation of visual function following argon laser photocoagulation in central serous retinopathy." Ophthalmic surgery and lasers **28**(8): 693-697.

The authors of previous studies on the role of photocoagulation for central serous chorioretinopathy (CSR) have based their deductions on the premise that a Snellen visual acuity of 6/6 is the end point

of recovery. It is now known that patients with a visual acuity of 6/6 may have defective contrast sensitivity indicative of a visual function deficit. The present study was a prospective, controlled, and randomized evaluation of patients suffering from their first attack of CSR, in which contrast sensitivity was used to determine the effectiveness of argon laser photocoagulation as compared with more conservative treatment. Although long-term studies are necessary, the results of this study showed that while photocoagulation increases the rate of recovery for visual acuity, it is also linked with significant loss and slower recovery of contrast sensitivity.

Kianersi, F. and F. Fesharaki (2008). "Effects of propranolol in patients with central serous chorioretinopathy." Journal of research in medical sciences **13**(3): 103-107.

Background: Central serous retinopathy (CSR) is a frequent disease often involves healthy men and causes visual disturbances. This study was undertaken to investigate the effects of propranolol tablet on improvement of CSR in referral cases to Farabi and Feiz hospitals in Isfahan in 2003-2004. Methods: This was a double-blind randomized controlled clinical trial. Patients with CSR referred to Farabi and Feiz hospitals were randomly divided into 2 groups: treatment group and control group, each one included 30 patients. Treatment group took propranolol tablets 20 mg twice a day, and control group took placebo tablets. Patients were examined weekly until complete improvement. Means of duration of disease and final visual acuity on the basis of lag mar were compared by t-test. Relative frequency of final visual acuity of 10/10 in the two groups was compared using chisquare tests. Results: Two groups were comparable with respect to age, sex, involved eye, psychological tensions and clinical findings. The course of improvement in treatment group was 62 ± 29 days and in control group was 89 ± 44 days (P < 0.05). Means of final visual acuities were not significantly different between the two groups (0.98 ± 0.13 log mar in treatment group compared to 0.97 ± 0.18 log mar in control group). Relative frequency of vision < 10/10 was 30% in control group and 3.3% in treatment group (P < 0.01). Conclusion: Duration of disease and need to laser therapy in patients with CSR were decreased by the use of propranolol but it had no effect on the amount of final vision. Because the patients are mostly in the years of active life, propranolol use is recommended for these patients.

Kim, J. A., et al. (2015). "Comparison of Choroidal Hyperpermeability Change after Photodynamic Therapy and Ranibizumab for Chronic Central Serous Chorioretinopathy." J korean ophthalmol soc **56**(2): 205-212.

PURPOSE: To compare changes in choroidal hyperpermeability after half-energy photodynamic therapy (PDT) and intravitreal ranibizumab in the treatment of chronic central serous chorioretinopathy (CSC). METHODS: Post-hoc analysis was performed in a randomized, controlled trial comparing half-energy PDT versus intravitreal ranibizumab for chronic CSC; during the experiments, the other treatment was available for salvage treatment if the original was unsuccessful at 3 months. A commercially available image analysis program (Adobe(R) Photoshop(R) CS6 [Adobe Systems, Inc., San Jose, CA]) was used for quantification of change in choriodal hyperpermeability on indocyanine green angiography after half-energy PDT or three consecutive intravitreal injections of ranibizumab. Post-treatment images were subtracted from pre-treatment images after adjustments were made to create images depicting the change in choroidal hyperpermeability with treatment. Integrated gray scale values per area in this image were used for analysis of change in choroidal hyperpermeability. RESULTS: The calculated change in choroidal hyperpermeability was significantly greater in the half-energy PDT group (17.36 +/- 8.74) than in the ranibizumab group (6.78 +/- 5.03) (p < 0.001). All eyes in the half-energy PDT group showed complete resolution of subretinal fluid, and no significant difference in change of choroidal hyperpermeability was found in eyes that received half-energy PDT as primary or salvage

treatment. In the ranibizumab-treated group, subretinal fluid resolution was accomplished in 5 eyes, and these eyes showed a significantly larger decrease in choroidal hyperpermeability when compared with eyes showing poor response (10.31 +/- 4.00 vs. 2.74 +/- 2.16, p = 0.005). In the successfully treated eyes with ranibizumab, there was no significant difference in choroidal hypopermeability change when compared to half-energy PDT (p = 0.124). CONCLUSIONS: Using our novel method of analysis of change in choroidal hyperpermeability following treatment for chronic CSC, greater change was found in eyes with good response, and the superior outcome of half-energy PDT over ranibizumab may be attributed to greater influence on choroidal hyperpermeability.

Kim, M., et al. (2013). "Intravitreal ranibizumab for acute central serous chorioretinopathy." Ophthalmologica. Journal international d'ophtalmologie [International journal of ophthalmology] **229**(3): 152-157.

BACKGROUND/AIMS: To evaluate the effectiveness of intravitreal ranibizumab injection (IVRI) for acute central serous chorioretinopathy (CSC). METHODS: Patients with symptomatic CSC of less than 3 months were prospectively recruited. Patients (n = 20/group) were randomly assigned to IVRI (0.5 mg/0.05 ml) or observation and followed for 6 months. logMAR best-corrected visual acuity (BCVA), fluorescein angiography, indocyanine angiography, and central foveal thickness (CFT) were assessed at baseline and at regular follow-ups. RESULTS: All patients had increased BCVA, decreased CFT, and resolution of the neurosensory detachment. Complete resolution of neurosensory retinal detachment required more time in the observation group (13.0 ± 3.1 vs. 4.2 ± 0.9 weeks; p < 0.001). Mean BCVA and mean CFT improved significantly in both groups, but the changes were not significantly different between groups at 6 months. CONCLUSIONS: IVRI for acute CSC might hasten resolution of neurosensory detachment compared to observation alone. At 6 months, BCVA and CFT did not differ between IVRI and observation groups. Further studies are required to determine the long-term benefits of IVRI.

Klatt, C., et al. (2011). "Selective retina therapy for acute central serous chorioretinopathy." British journal of ophthalmology **95**(1): 83-88.

Aims To evaluate selective retina therapy (SRT) as a treatment of acute central serous chorioretinopathy. Methods 30 eyes of 30 patients with central serous chorioretinopathy of at least a 3 months' duration were recruited. 14 eyes were randomised to an SRT group (Q-switched neodymium-doped yttrium lithium fluoride (Nd:YLF) laser, wavelength 527 nm, t =1.7 mu s, energy 100-370 mu J, spot diameter 200 mu m, pulse repetition rate 100 Hz,) and 16 eyes to a control group. After 3 months of follow-up, patients in the control group with persistence of subretinal fluid (SRF) were allocated to a cross-over group, treated with SRT and followed up for further 3 months. The main outcome measures were change of best-corrected Early Treatment Diabetic Retinopathy Study visual acuity (BCVA) and SRF. Results At 3 months of follow-up, the mean (SD) improvement of BCVA was significantly greater after SRT than in the control group: 12.7 (7.2) versus 6.3 (8.9) letters (p=00.04). SRF had decreased significantly more after SRT as compared with that the control group: 203 (136) mu m versus 41 (150) mu m (p=0.005). In eight eyes allocated to the cross-over group, the mean BCVA had increased during 3 months of follow up before SRT by 1.4 (5.2) letters and continued to increase during 3 months following SRT by 7.4 (6.3) letters, while SRF increased by 39.5 (160.2) mu m before SRT and decreased by 151.5 (204.9) mu m after SRT. In six of the eight eyes, SRF had completely resolved 3 months after SRT. Conclusions SRT appears to expedite functional recovery and the re-absorption of SRF as compared with that in untreated controls. A larger prospective, randomised phase 3 confirmative patient study is warranted.

Kretz, F. T. A., et al. (2015). "Randomized clinical trial to compare micropulse photocoagulation versus half-dose verteporfin photodynamic therapy in the treatment of central serous chorioretinopathy." Ophthalmic surgery lasers and imaging retina **46(8)**: 837-843.

Background and Objective: To evaluate subthreshold diode-laser micropulse (SDM) versus half-dose verteporfin photodynamic therapy (hd- PDT) in central serous chorioretinopathy (CSC). Patients and Methods: 62 eyes of 62 patients were prospectively followed for changes in fluorescein angiography (FA), fundus autofluorescence (FAF), central macular thickness (CMT), best-corrected visual acuity (BCVA), and contrast visual acuity (CVA) after SDM (n = 20) or hdPDT (n = 24). CSC observation served as control group (n = 18). Result(s): Both treatment groups (60% SDM vs. 66.7% hdPDT) showed significant improvement in reduction of leakage activity compared to the control group (37.5%) at 16 weeks. CMT decreased by 69.7 mum (SDM), 109.8 mum (hdPDT), and 89 mum (control). BCVA improved by +6.7 (SDM group), +8.5 (hdPDT), and +1.5 ETDRS letters (control). CVA was best improved in the hdPDT group. No secondary RPE alterations could be detected by FAF after any intervention. Conclusion(s): In comparison to the control group, hdPDT and SDM resulted in reduced leakage activity in FA and enhanced photopic and scotopic visual acuity in patients with CSC.

Kustryn, T., et al. (2022). "Photodynamic Therapy with Chlorin e6 Derivative for Chronic Central Serous Chorioretinopathy (Pilot Study)." Journal of Ocular Pharmacology and Therapeutics **38(7)**: 505-512.

Purpose: To evaluate the use of photodynamic therapy (PDT) with chlorin e6 (Ce6) derivative in patients with chronic central serous chorioretinopathy (CSC). Method(s): Participants in this interventional, single-center clinical pilot study included 39 patients (39 eyes) with chronic CSC. Primary objectives were to assess safety and change in decimal best-corrected visual acuity (BCVA) during the 12-month follow-up. Secondary objectives were to evaluate change in central retinal thickness (CRT) in fovea area, maximal height of subretinal fluid (SRF), subfoveal choroidal thickness (SFCT) on spectral-domain optical coherence tomography, and number of treatments. Result(s): There were no systemic adverse events and ocular side effects. Mean decimal BCVA showed a significant increase from 0.49 +/- 0.25 to 0.63 +/- 0.28 (P = 0.001), mean CRT and maximal height of SRF decreased significantly from 335 +/- 95 to 219 +/- 69 mum and from 149 +/- 84 to 32 +/- 86 mum respectively (P = 0.001), comparing baseline and month 12. There was no significant difference between mean SFCT before PDT and at month 12. During all follow-up periods mean number of PDT treatments was 1.3 +/- 0.7. Complete SRF resolution was observed in 82% (32 eyes) during observation period. Conclusion(s): Results of this pilot study demonstrate that PDT with Ce6 derivative is a safe method to treat eyes with chronic CSC. PDT with Ce6 derivative is the treatment option for chronic CSC. Further randomized controlled studies with a larger sample size and longer periods of follow-up are needed to assess the effectivity and safety of PDT with Ce6 derivative in chronic CSC. Copyright © 2022, Mary Ann Liebert, Inc., publishers 2022.

Lai, T. Y. Y., et al. (2018). "EFFICACY AND SAFETY OF RANIBIZUMAB FOR THE TREATMENT OF CHOROIDAL NEOVASCULARIZATION DUE TO UNCOMMON CAUSE Twelve-Month Results of the MINERVA Study." Retina-the Journal of Retinal and Vitreous Diseases **38**(8): 1464-1477.

Purpose: To evaluate the efficacy and safety of ranibizumab 0.5 mg in adult patients with choroidal neovascularization because of an uncommon cause enrolled in the 12-month MINERVA study. Methods: In this Phase III, double-masked study, adult ($ 18 years) patients (N = 178) were randomized 2: 1 to receive either ranibizumab (n = 119) or sham (n = 59) at baseline and, if needed, at Month 1 and open-label individualized ranibizumab from Month 2. Bestcorrected visual acuity change from baseline to Month 2 (primary endpoint) and Month 12, treatment exposure, and safety

over 12 months were reported. Subgroup analysis was conducted on five predefined choroidal neovascularization etiologies (angioid streak, postinflammatory, central serous chorioretinopathy, idiopathic, and miscellaneous). Results: Ranibizumab showed superior efficacy versus sham from baseline to Month 2 (adjusted least-squares mean best-corrected visual acuity: + 9.5 vs. 20.4 letters; P, 0.001). At Month 12, the mean best-corrected visual acuity change was + 11.0 letters (ranibizumab) and + 9.3 letters (sham). Across the 5 subgroups, the treatment effect ranged from + 5.0 to + 14.6 letters. The mean number of ranibizumab injections was 5.8 (ranibizumab arm) with no new ocular or nonocular adverse events. Conclusion: Ranibizumab 0.5 mg resulted in clinically significant treatment effect versus sham at Month 2. Overall, ranibizumab was effective in treating choroidal neovascularization of various etiologies with no new safety findings.

Lanzetta, P., et al. (2008). "Nonvisible subthreshold micropulse diode laser (810 nm) treatment of central serous chorioretinopathy. A pilot study." European journal of ophthalmology **18(6)**: 934-940.

Purpose. To verify the efficacy of nonvisible micropulse diode laser irradiation in the treatment of central serous chorioretinopathy (CSC). Methods. Twenty-two patients with CSC for a total of 24 eyes with a disease duration longer than 3 months were included in a prospective study. Patients underwent Early Treatment Diabetic Retinopathy Study visual acuity (VA) examination, dilated ophthalmoscopy, fluorescein angiograppy, and optical coherence tomography before treatment and during follow-up. Treatment with a micropulse diode laser was given with a duty cycle of 15%. Multiple spots were placed over and adjacent to the area of retinaf pigment epithelium leak or decompensation. Results. Mean follow-up was 14 months (range 3-36 months). Powers used ranged from 1 to 2 W (mean 1.35 W). Mean number of spots was 275 (range 90-400). Fourteen eyes were treated once, nine eyes received two to three treatmonts, and one eye had five treatments during a follow-up of 3 years. Subretinal fluid was resolved or improved in'two third of cases 1 month after laser treatment, and in three-quarters at the end of follow-up. Mean retinal thickness was 328 mum, 197 mum, and 168, mum before, 1 month after irradiation, and at the end of follow-up, respectively. No evidence of RPE or retinal changes due to laser treatment were discernible in most of the eyes. Median VA was 20/ 32 (range 20/100-20/20) before treatment and 20/25 (range 20/200-20/20) at the end-of the follow-up. Conclusions. Nonvisible micropulse diode laser may have efficacy in the treatment of CSC. A randomized study with larger series is needed. © Wichtig Editore, 2008.

Lee, J.-Y., et al. (2021). "The Effect of Selective Retina Therapy with Automatic Real-Time Feedback-Controlled Dosimetry for Chronic Central Serous Chorioretinopathy: A Randomized, Open-Label, Controlled Clinical Trial." Journal of clinical medicine **10**(19).

This prospective randomized controlled trial evaluated the safety and efficacy of real-time feedback-controlled dosimetry (RFD)-guided selective retina therapy (SRT) in chronic central serous chorioretinopathy (CSC). Forty-four participants with chronic CSC were included and randomly assigned to the control group or SRT group. The SRT laser system with RFD-guidance was applied to cover the entire leakage area. If SRF remained at the 6-week follow-up visit, re-treatment and rescue SRT was performed for the SRT group and crossover group, respectively. The rate of complete resolution of subretinal fluid (SRF), mean SRF height, and mean retinal sensitivity were compared between the two groups at 6-weeks post-treatment. The complete SRF resolution rate in all SRT-treated eyes was evaluated at 12-weeks post-treatment. The rate of complete SRF resolution was significantly higher in the SRT group (63.6%) than in the control group (23.8%) at 6-weeks post-treatment ($p = 0.020$). The mean SRF height at 6 weeks after SRT was significantly lower in the SRT group ($p = 0.041$). Overall, SRT-treated eyes showed complete SRF resolution in 70.3% of eyes at 12-weeks post-treatment. RFD-guided SRT was safe and effective to remove SRF in chronic CSC patients during the 3-month follow-up period.

Lim, J. W., et al. (2010). "The Effect of Intravitreal Bevacizumab in Patients with Acute Central Serous Chorioretinopathy." Korean Journal of Ophthalmology **24**(3): 155-158.

Purpose: To evaluate the effect of intravitreal bevacizumab injection (IVBI) in acute central serous chorioretinopathy (CSC) patients.Methods: Patients with acute CSC received IVBI (1.25 mg/0.05 mL) or observation by randomization. Twelve eyes in each group completed 6 months of regular follow-up and were ultimately included in this study. Each patient was assessed using best corrected visual acuity measurements, fluorescein angiography, and optical coherence tomography at baseline and had regular follow-ups after treatment.Results: All patients showed improvements in visual acuity and fluorescein angiographic leakage and had resolution of their neurosensory detachment following treatment. There were no significant differences in visual acuity, central retinal thickness, or remission duration between the IVBI group and the control group at baseline or after treatment ($p > 0.05$).Conclusions: Intravitreal bevacizumab showed no positive effect in acute CSC patients compared to the observation group, and there were no adverse effects of treatment. Further investigation will be helpful to understand this therapy in patients with CSC.

Lotery, A., et al. (2020). "Eplerenone for chronic central serous chorioretinopathy in patients with active, previously untreated disease for more than 4 months (VICI): a randomised, double-blind, placebo-controlled trial." Lancet **395**(10220): 294-303.

Background In chronic central serous chorioretinopathy (CSCR), fluid accumulates in the subretinal space. CSCR is a common visually disabling condition that develops in individuals up to 60 years of age, and there is no definitive treatment. Previous research suggests the mineralocorticoid receptor antagonist, eplerenone, is effective for treating CSCR; however, this drug is not licensed for the treatment of patients with CSCR. We aimed to evaluate whether eplerenone was superior to placebo in terms of improving visual acuity in patients with chronic CSCR. Methods This randomised, double-blind, parallel-group, multicentre placebo-controlled trial was done at 22 hospitals in the UK. Participants were eligible if they were aged 18-60 years and had had treatment-naive CSCR for 4 months or more. Patients were randomly assigned (1:1) to either the eplerenone or the placebo group by a trial statistician through a password-protected system online. Allocation was stratified by best-corrected visual acuity (BCVA) and hospital. Patients were given either oral eplerenone (25 mg/day for 1 week, increasing to 50 mg/day for up to 12 months) plus usual care or placebo plus usual care for up to 12 months. All participants, care teams, outcome assessors, pharmacists, and members of the trial management group were masked to the treatment allocation. The primary outcome was BCVA, measured as letters read, at 12 months. All outcomes apart from safety were analysed on a modified intention-to-treat basis (participants who withdrew consent without contributing a post-randomisation BCVA measurement were excluded from the primary analysis population and from most secondary analysis populations). The trial is registered with ISRCTN, ISRCTN92746680, and is completed. Findings Between Jan 11, 2017, and Feb 22, 2018, we enrolled and randomly assigned 114 patients to receive either eplerenone (n=57) or placebo (n=57). Three participants in the placebo group withdrew consent without contributing a post-randomisation BCVA measurement and were excluded from the primary outcome analysis population. All patients from the eplerenone group and 54 patients from the placebo group were included in the primary outcome. Modelled mean BCVA at 12 months was 79.5 letters (SD 4.5) in the placebo group and 80.4 letters (4.6) in the eplerenone group, with an adjusted estimated mean difference of 1.73 letters (95% CI -1.12 to 4.57; p=0.24) at 12 months. Hyperkalaemia occurred in eight (14%) patients in each group. No serious adverse events were reported in the eplerenone group and three unrelated serious adverse events were reported in the placebo group (myocardial infarction [anticipated], diverticulitis [unanticipated], and metabolic surgery [unanticipated]).

Interpretation Eplerenone was not superior to placebo for improving BCVA in people with chronic CSCR after 12 months of treatment. Ophthalmologists who currently prescribe eplerenone for CSCR should discontinue this practice. Copyright (C) 2020 The Author(s). Published by Elsevier Ltd.

Loya, H., et al. (2019). "Effect of altering the regime of oral rifampicin therapy in the treatment of persistent central serous chorioretinopathy." Pakistan journal of medical sciences **35**(6): 1687-1690.

Objective: To study the effect of reducing the duration of rifampicin therapy in the treatment of Chronic Central Serous Chorioretinopathy. Methods: This is interventional study conducted in Layton Rahmatullah Benevolent Trust, Free Base Eye Hospital Korangi, Karachi from February 2017-December 2018. This randomized controlled comparative study included two groups, Groups-A comprised of 48 eyes of 40 cases with Chronic Central Serous Chorioretinopathy who were given reduced dose of oral rifampicin i.e. 600mg for one month, and Group-B consisted of 43 eyes of 40 controls with Chronic Central Serous Chorioretinopathy who were given reduced dose of oral rifampicin i.e. 300mg once daily for three months as previously stated in literature. To access the effect of therapy in both the groups, pre-treatment visual acuity on the logMAR and Optical Coherent Tomography (OCT, Heidelberg spectralis) for CMT were performed and repeated on the 1st and 3rd month post-treatment. Patients were also followed for 6 months to access any recurrence. Results: On comparing the two groups, Group-A had improvement in VA and CMT after one month therapy of Rifampicin, Pre-treatment mean VA in Group-A was 0.85 ± 0.19 as compared to the pre-treatment mean VA in Group-B i.e. 0.74+/-0.208, while the pre-treatment mean CMT was 609.0 ± 178.29 μm in Group-A, and 600.0 +/-155.09 μm in Group-B respectively. After 1 month of therapy, the visual status, and CMT in Group-A was 0.29+/-0.21 and 311.6 +/-89.9, while Group-B, VA was 0.598 +/-0.23 (p value 0.001%) and CMT was 512.30 +/-148.37 (p-value 0.001%). Rifampicin was continued in Group-B till three months, and patients were re-accessed but there was no difference in VA and CMT statically. During the 3rd and 6th months of follow up no relapses were reported. Conclusion: This comparative study showed that the group receiving oral rifampicin 600mg for one month showed better outcome at one month and third month than the group receiving oral rifampicin at a dose of 300mg once daily for three months. This gives a better compliance and lower the risk of drug induced side effects.

Missotten, T. O. A. R., et al. (2021). "A randomized clinical trial comparing prompt photodynamic therapy with 3 months observation in patients with acute central serous chorioretinopathy with central macular leakage." European journal of ophthalmology **31(3)**: 1248-1253.

Purpose: The purpose of the study was to demonstrate whether photodynamic therapy in patients with acute central serous chorioretinopathy, with the leakage point within one optic disk diameter from the fovea, can be safely deferred. Method(s): A single-center, randomized, controlled trial was conducted. Patients were randomized to photodynamic therapy within a week after presentation (Group I, 26 patients) or observation during 3 months (Group II, 26 patients). If leakage or subretinal fluid was observed during any control visit, photodynamic therapy was performed (again) within a week. Primary Outcome: Primary outcome was change of visual acuity (Early Treatment Diabetic Retinopathy Study) after 12 months. Secondary outcomes were visual acuity, central foveal thickness, metamorphopsia, and color discrimination. Result(s): Photodynamic therapy procedures: group I, 26 at baseline, 2 retreatments at 3 months; group II, 10 at 3 months, 1 at 6 months (2 subjects refusing treatment), 2 retreatments at 6 months. At 12 months, mean visual acuity of all patients had improved by 6.5 letters ($P < 0.001$), mean central foveal thickness was 172 microm less ($P < 0.001$). After photodynamic therapy, visual acuity recovered faster and metamorphopsia significantly improved (3 months, $P < 0.001$). Differences between groups at 12

months were not significant. Conclusion(s): The (intended) number of photodynamic therapy (re)treatments in group II (n = 15) was 46% less than in group I (n = 28). Visual acuity and central foveal thickness at 12 months were similar. Therefore, the preferred management of acute central serous chorioretinopathy at presentation appears to be observation for 3 months. Copyright © The Author(s) 2020.

Nongrem, G., et al. (2021). "Effect of short-term meditation training in central serous chorioretinopathy." Indian journal of ophthalmology **69**(12): 3559-3563.

Purpose: Stress and Type A personality are established risk factors for the development of central serous chorioretinopathy (CSC). Meditation is known to have a positive effect on reducing stress levels. This study aimed to assess the effect of short-term meditation training in patients of CSC.Methods: A pilot study was conducted where 40 patients diagnosed with acute and non-resolving CSC were randomly assigned to either of two groups - meditation training and routine care (without meditation). The primary outcome measure was time to resolution of CSC based on optical coherence tomography and fluorescein angiography. Secondary outcome measures were changes in anxiety score (State-Trait Anxiety Inventory [STAI] scores) and blood pressure. The patients were followed up for a minimum period of 4 months.Results: Twenty cases were included in each group. The demographic pattern, baseline swept-source optical coherence tomography parameters, and STAI scores were similar in both groups. The time to disease resolution was 9.4 ± 4.22 weeks in the meditation group and 19.5 ± 2.79 weeks in the nonmeditation group (P < 0.001). At 4 months, CSC had failed to resolve in 60% of patients with routine care compared with 8% in cases following short-term meditation training. STAI scores showed a reduction in stress levels in the meditation group. Furthermore, statistically significant improvement in systolic and diastolic blood pressures was also observed following meditation training.Conclusion: Short-term meditation training may be a useful approach in the management of patients with CSC as it tends to reduce stress and prehypertension, and promotes earlier resolution of the condition. However, patient's motivation to complete and pursue the meditation training is a significant barrier.

Oh, J. R., et al. (2021). "Evaluation of the Safety and Efficacy of Selective Retina Therapy Laser Treatment in Patients with Central Serous Chorioretinopathy." Korean Journal of Ophthalmology **35**(1): 51-63.

Purpose: To assess the safety and efficacy of selective retina therapy (SRT) using a Q-switched neodymium-doped yttriumlithium fluoride laser with feedback systems in patients with idiopathic central serous chorioretinopathy (CSC).Methods: This randomized clinical trial enrolled patients having at least 3-month symptom of CSC. From month 3 visit, allsubjects in both groups were eligible for SRT retreatment if they showed persistent or recurrent subretinal fluid (SRF). The primaryoutcome was complete resolution of SRF by optical coherence tomography at 3 months after treatment. The secondaryoutcomes were changes in SRF, central macular thickness (CMT) and best-corrected visual acuity at the 1-, 3-, and 6-monthexaminations.Results: Sixty-eight CSC patients were included (SRT, 31; control, 37). After 1 and 3 months, complete resolution of SRF wasachieved in 25.8% and 54.8% of SRT group and 17.6% and 35.1% of controls. The differences were not statistically significant(p = 0.424 and p = 0.142, respectively). However, mixed model for repeated measures analyses showed that the reduction ofSRF and CMT were observed earlier in SRT group than in the sham group (least squares mean difference, -59.7 μm; 95% confidenceinterval, -98.2 to -21.2; p = 0.0029; least squares mean difference -67.0 μm; 95% confidence interval, -104.8 to -29.2;p = 0.0007, respectively). Significant reduction of SRF (â‰¥50% reduction from baseline) was more frequently observed in SRTgroup (80.6%) than the sham group (44.1%) at month 1 (p = 0.007). Early reduction of SRF and CMT was more abundant inSRT group with symptom duration less than 6 months. Treatment related serious adverse events

were not observed.Conclusions: SRT using a Q-switched neodymium-doped yttrium lithium fluoride laser with feedback system was safe in thistrial and effective for early resolution of SRF in the CSC patients. Early intervention with SRT can be a safe alternative for patientswith acute symptomatic CSC.

Park, D. G., et al. (2021). "Optimal fluence rate of photodynamic therapy for chronic central serous chorioretinopathy." British journal of ophthalmology **105**(6): 844-849.

AIMS: To investigate the lowest effective fluence rate of photodynamic therapy (PDT) for treating chronic central serous chorioretinopathy (CSC). METHODS: Fifty-one eyes of 51 patients with chronic CSC were randomly treated with 30% (n=15), 40% (n=16) or 50% (n=17) of the standard-fluence rate of PDT and followed up for 12 months. The success rate, recurrence rate, mean best-corrected visual acuity (BCVA), central foveal thickness (CFT), subfoveal choroidal thickness (SFCT), integrity of the outer retinal layer and complications were evaluated at baseline and at the follow-up periods after PDT. RESULTS: The rate of complete subretinal fluid (SRF) resolution in the 30%-fluence, 40%-fluence and 50%-fluence groups was 60.0%, 81.2% and 100.0%, respectively, at 3 months (p=0.009), and 80.0%, 94.0% and 100.0%, respectively, at 12 months (p=0.06). The recurrence rate in the 50%-fluence group was lower than that in the 30%- and 40%-fluence groups at 12 months (30% vs 50%, 40% vs 50%; p=0.002, p=0.030, respectively (log-rank test)). The mean BCVA improved significantly 12 months after PDT only in the 40%- and 50%-fluence groups (p=0.005, p=0.003, respectively). Mean CFT and SFCT decreased significantly at 12 months in the three groups. The rate of complications did not differ significantly among the three groups. CONCLUSIONS: A 50%-fluence rate of PDT seems to be the most effective for treating chronic CSC, considering the low recurrence rate and high rate of complete SRF resolution, compared with other low-fluence PDT. TRIAL REGISTRATION NUMBER: NCT01630863.

Pfau, M., et al. (2021). "Estimation of current and post-treatment retinal function in chronic central serous chorioretinopathy using artificial intelligence." Scientific reports **11**(1): 20446.

Refined understanding of the association of retinal microstructure with current and future (post-treatment) function in chronic central serous chorioretinopathy (cCSC) may help to identify patients that would benefit most from treatment. In this post-hoc analysis of data from the prospective, randomized PLACE trial (NCT01797861), we aimed to determine the accuracy of AI-based inference of retinal function from retinal morphology in cCSC. Longitudinal spectral-domain optical coherence tomography (SD-OCT) data from 57 eyes of 57 patients from baseline, week 6-8 and month 7-8 post-treatment were segmented using deep-learning software. Fundus-controlled perimetry data were aligned to the SD-OCT data to extract layer thickness and reflectivity values for each test point. Point-wise retinal sensitivity could be inferred with a (leave-one-out) cross-validated mean absolute error (MAE) [95% CI] of 2.93 dB [2.40-3.46] (scenario 1) using random forest regression. With addition of patient-specific baseline data (scenario 2), retinal sensitivity at remaining follow-up visits was estimated even more accurately with a MAE of 1.07 dB [1.06-1.08]. In scenario 3, month 7-8 post-treatment retinal sensitivity was predicted from baseline SD-OCT data with a MAE of 3.38 dB [2.82-3.94]. Our study shows that localized retinal sensitivity can be inferred from retinal structure in cCSC using machine-learning. Especially, prediction of month 7-8 post-treatment sensitivity with consideration of the treatment as explanatory variable constitutes an important step toward personalized treatment decisions in cCSC.

Pichi, F., et al. (2017). "Comparison of two mineralcorticosteroids receptor antagonists for the treatment of central serous chorioretinopathy." Int Ophthalmol **37**(5): 1115-1125.

PURPOSE: To evaluate the effect of oral spironolactone and eplerenone, two specific antagonists of the mineralocorticoid receptor, in central serous chorioretinopathy (CSCR). METHODS: In this prospective, placebo-controlled trial, sixty patients with persistent CSCR were assigned to three treatment group. Twenty patients in Group 1 were treated with 25 mg of spironolactone (Aldactone; Pfizer) for 1 week, then increased to 50 mg for the following 3 weeks, then shifted to eplerenone 50 mg for 1 month. Twenty patients in Group 2 were treated with 25 mg of eplerenone (Inspra; Pfizer) for 1 week, then increased to 50 mg for the following 3 weeks, and then shifted to spironolactone 50 mg for 1 month. Twenty patients in Group 3 were treated with 1 placebo control tablet for 1 week, then increased to two tablets for the following 3 weeks, and then shifted to spironolactone 50 mg for 1 month. At the end of the second month, all the treatments were stopped, and the patients were followed for two additional months. Primary outcome measure was a change in BCVA at 1, 2, and 4 months. Secondary outcome was a change of >20 % in the size of SRF recorded with OCT at 1, 2, and 4 months of treatment. RESULTS: In terms of BCVA, treatment in Group 1 was effective from the first month (spironolactone, p value 0.01), and in Group 2 effective from the second month (shift to spironolactone, p value 0.004). Since the p value after the first month was 0.2 in Group 2, even with a larger sample, it would be difficult to see an efficacy of an eplerenone treatment after 1 month. As for the SRF, both in Group 1 and Group 2, both treatments were found to be equally effective after 1 month of administration (p values 0.004). At 4 months, only in Group 3, there was no statistical improvement of BCVA and SRF (p values 0.09 and 0.5). CONCLUSIONS: Spironolactone is statistically superior to eplerenone in improving BCVA of patients with CSCR, while both drugs can be considered equally effective in promoting the reabsorption of SRF.

Rahimy, E., et al. (2018). "A RANDOMIZED DOUBLE-BLIND PLACEBO-CONTROL PILOT STUDY OF EPLERENONE FOR THE TREATMENT OF CENTRAL SEROUS CHORIORETINOPATHY (ECSELSIOR)." Retina (Philadelphia, Pa.) **38**(5): 962-969.

PURPOSE: To evaluate the safety and effects of oral eplerenone in chronic central serous chorioretinopathy. METHODS: Prospective, randomized, double-blind, placebo-control study at a tertiary referral academic private practice. For a diagnosis of chronic central serous chorioretinopathy, patients must have had at least 3 months clinical follow-up demonstrating persistent symptoms, subfoveal fluid on spectral-domain optical coherence tomography, and <50% reduction in fluid thickness. Patients were randomized 2:1 (treatment:placebo) to receive eplerenone (25 mg daily for 1 week, then up to 50 mg daily for 8 weeks) or placebo once daily. RESULTS: Fifteen patients completed the study. Ten patients (15 eyes) were randomized into the eplerenone treatment arm, while the remaining 5 patients (6 eyes) received placebo. After 9 weeks of eplerenone therapy, mean logarithm of the minimal angle of resolution visual acuity improved from 0.394 (Snellen equivalent: 20/50) to 0.330 (20/43, P = 0.04). In the placebo group, the mean logarithm of the minimal angle of resolution visual acuity slightly decreased from 0.313 (20/41) to 0.342 (20/44) during the same period (P = 0.21). With respect to anatomic changes, mean maximal subretinal fluid height in the eplerenone group improved from 139.3 μm at baseline to 51.8 μm (P = 0.02), mean subfoveal fluid height improved from 121.4 μm to 29.4 μm (P = 0.01), and mean central subfield thickness improved from 366.2 μm to 283.7 μm (P = 0.02). In comparison with the placebo group, mean maximal subretinal fluid height worsened from 135.9 μm to 172.3 μm (P = 0.32), mean subfoveal fluid height worsened from 92.1 μm to 134.0 μm (P = 0.54), and mean central subfield thickness worsened from 345.0 μm to 380.0 μm (P = 0.37). No patients in either group experienced serious adverse events to result in treatment discontinuation. CONCLUSION: These findings suggest that oral eplerenone therapy is safe and potentially effective in the treatment of chronic central serous chorioretinopathy with persistent subretinal fluid.

Ratanasukon, M., et al. (2012). "High-dose antioxidants for central serous chorioretinopathy; the randomized placebo-controlled study." BMC ophthalmology **12**: 20.

BACKGROUND: To determine the efficacy of high-dose antioxidants in the acute stage of central serous chorioretinopathy (CSC). METHODS: This was a randomized placebo-controlled study. The patients with acute CSC (onset within 6 weeks) were randomized to receive either high-dose antioxidant tablets (study group A) or placebo tablets (control group B) for 3 months or until the complete resolution of subretinal fluid. After 3 months, additional treatment with laser or photodynamic therapy (PDT) was considered if any fluorescein leakage persisted. The outcomes measured were the changes in visual acuity (VA) and central macular thickness (CMT), the number of patients with subretinal fluid at each follow-up time, the number of patients with fluorescein leakage at the end of the 3rd month and patients who received additional treatments. RESULTS: Fifty-one of 58 patients (88%) completed the follow-up criteria. The baseline demographic data were comparable in both groups. At the end of the 3rd month, the VA and CMT showed no statistical difference between the groups but the patients in group A has less fluorescein leakage and additional treatments than in group B ($p = 0.027$ and 0.03). CONCLUSION: The high-dose antioxidants for acute CSC did not show any benefits in VA and CMT. However, the drugs might decrease the chance for fluorescein leakage and additional treatments at the end of the 3rd month.

Robertson, D. M. and D. Ilstrup (1983). "Direct, indirect, and sham laser photocoagulation in the management of central serous chorioretinopathy." American journal of ophthalmology **95**(4): 457-466.

The role of argon laser photocoagulation in the management of central serous choriorectinopathy was evaluated in a prospective randomized study of eyes with leaks smaller than 250 microns in diameter in the early frames of the angiogram. Eyes were assigned to Group A when the leak was located in the papillomacular bundle or within 500 microns of the capillary-free zone and to Group B when the leakage site was located outside the papillomacular bundle and more than 500 microns from the capillary-free zone. Thirty eyes in Group A underwent either a sham argon laser treatment or a real argon laser treatment directed to the pigment epithelium under the elevated retina at a site remote from the site of fluorescein dye leakage (indirect laser photocoagulation). Twelve eyes in Group B underwent either a laser treatment directed to the site of fluorescein dye leakage (direct laser photocoagulation) or indirect laser photocoagulation. Compared with indirect photocoagulation, direct laser photocoagulation shortened the duration of central serous chorioretinopathy by approximately two months, a statistically significant difference. When compared to sham treatment, indirect photocoagulation did not significantly alter the duration of central serous chorioretinopathy. During an 18-month interval, the recurrence rate in the eyes treated with sham and indirect laser photocoagulation was 34%, whereas no recurrences were observed in the eyes treated with direct photocoagulation. There were no complications from photocoagulation in any of the eyes.

Roisman, L., et al. (2013). "Micropulse diode laser treatment for chronic central serous chorioretinopathy: a randomized pilot trial." Ophthalmic surgery, lasers & imaging retina **44**(5): 465-470.

Background and Objective: To evaluate 810-nm subthreshold diode micropulse (SDM) laser in patients with chronic central serous chorioretinopathy (CSC).Patients and Methods: Prospective, randomized, double-blind, sham-controlled pilot trial. Patients were randomized to SDM laser treatment (group 1) or sham procedure (group 2). Primary outcome measure was change in best corrected visual acuity (BCVA); secondary outcome was central macular thickness after 3 months. Laser treatment was performed along the detached area. At the 3-month visit, all patients were evaluated for re-treatment if they met re-treatment criteria.Results: Fifteen patients were included in

this study: five patients in the sham group and 10 in the treatment group. At 3 months, BCVA was significantly enhanced in the treatment group (P = .006) compared with the sham group (P = .498). All patients from the sham group needed treatment after 3 months. An improvement in central macular thickness and leakage on fluorescein angiography was noted in all treated patients (in both groups).Conclusion: In this limited-size, short-term exploratory study, SDM laser was effective in treating chronic CSC. There was no evidence of retinal damage induced by treatment.

Russo, A., et al. (2017). "Comparison of half-dose photodynamic therapy and 689 nm laser treatment in eyes with chronic central serous chorioretinopathy." Graefe's archive for clinical and experimental ophthalmology: 1-8.

Purpose: To compare visual and anatomical outcomes between half-dose photodynamic therapy (hd-PDT) and 689 nm laser therapy (689-LT) in chronic central serous chorioretinopathy (CSC). Methods: Forty eyes of 40 patients with symptomatic chronic CSC were randomized in a 1:1 ratio to receive either hd-PDT or 689-LT delivering 95 J/cm2 via an intensity application of 805 mW/cm2 over 118 s. Best-corrected visual acuity (BCVA) and spectral-domain optical coherence tomography findings were compared between the two treatment groups. Results: Mean CSC duration was 17.1 +/- 6.66 weeks and 18.7 +/- 7.46 weeks in the hd-PDT and 689-LT groups respectively. Both groups showed significant BCVA improvements, as well as reductions in central retinal and subfoveal choroidal thickness. Although hd-PDT led to a faster reduction in central retinal thickness, no significant differences were recorded between groups for any other measured parameter at any time point. Complete photoreceptor recovery was observed in eight and seven eyes in the hd-PDT and 689-LT groups respectively. Conclusions: Both hd-PDT and 689-LT were effective at treating chronic CSC. Further studies are warranted to evaluate long-term safety and efficacy. Copyright © 2017 Springer-Verlag Berlin Heidelberg

Sawa, M., et al. (2014). "Effects of a lutein supplement on the plasma lutein concentration and macular pigment in patients with central serous chorioretinopathy." Investigative ophthalmology & visual science **55**(8): 5238-5244.

PURPOSE: To investigate the effects of lutein supplementation on plasma lutein concentrations and the macular pigment optical density (MPOD) in central serous chorioretinopathy (CSC). METHODS: In this double-masked placebo-controlled study, 20 patients received lutein 20 mg/d and 19 received placebo. The plasma lutein concentration and MPOD using autofluorescence spectrometry (density unit, DU) were measured at baseline and 1 and 4 months. RESULTS: The mean plasma lutein concentrations and MPOD values in the lutein and control groups, respectively, were 91.5 and 78.2 ng/mL and 0.444 and 0.437 DU at baseline; 204.9 and 79.3 ng/mL and 0.460 and 0.442 DU at 1 month; and 228.0 and 78.4 ng/mL and 0.441 and 0.421 DU at 4 months. The plasma concentration in the lutein group was significantly higher than in controls at 1 and 4 months (P < 0.0001 for both comparisons); however, the MPOD values did not differ significantly between groups at 1 (P = 0.479) or 4 months (P = 0.883). In patients with a plasma lutein concentration below the mean level in 20 age-matched healthy subjects (mean 105.3 ng/mL; n = 13 in lutein group, n = 15 in control group), the control MPOD values significantly (P = 0.0430) decreased at 4 months (mean baseline, 0.437 DU; 4 months, 0.404 DU). The MPOD in the lutein group remained at the baseline level (mean baseline, 0.426 DU; 4 months, 0.438 DU) (P = 0.6542). CONCLUSIONS: The MPOD did not increase in patients with CSC with short-term lutein supplementation; however, among patients with low plasma lutein, supplemental lutein prevented a decline in MPOD that was observed in control subjects (www.umin.ac.jp/ctr number, UMIN000005849).

Schaal, K. B., et al. (2009). "Intravitreal bevacizumab for treatment of chronic central serous chorioretinopathy." European journal of ophthalmology **19(4)**: 613-617.

Purpose. To evaluate the short-term safety and efficacy of intravitreal bevacizumab for the treatment of intraretinal or subretinal fluid accumulation secondary to chronic central serous chorioretinopathy (CSC). Methods. Twelve patients were treated with intravitreal injections of 2.5 mg bevacizumab at 6- to 8-week intervals until intraretinal or subretinal fluid resolved. Observation procedures were Early Treatment Diabetic Retinopathy Study best-corrected visual acuity (BCVA), ophthalmic examination, and optical coherence tomography (OCT), performed at 6- to 8- week intervals. Fluorescein angiography was performed at baseline visit and thereafter depending on clinical and OCT findings. Multivariate analysis of variance with repeated measures was used to calculate a statistical significance of change in BCVA and mean central retinal thickness, which were the main outcome measures. SAS statistical software was used for analyses. Results. Patients received 2+/-1 intravitreal injections of bevacizumab on average during a follow-up of 24+/-14 weeks. Mean BCVA increased by 2+/-2 lines; the change in BCVA (log-MAR) was significant (p<0.02). Mean central retinal thickness decreased significantly over follow-up (p<0.05), with 6 patients (50%) showing complete resolution of subretinal fluid. Conclusions. Anatomic and functional improvement following intravitreal bevacizumab injections suggest that vascular endothelial growth factor (VEGF) may be involved in fluid leakage in patients with chronic CSC. The results suggest a possible role for anti-VEGF agents in the treatment of chronic CSC. Further evaluation of intravitreal bevacizumab for chronic CSC in controlled randomized studies is warranted. © Wichtig Editore, 2009.

Schwartz, R., et al. (2017). "Eplerenone for chronic central serous chorioretinopathy-a randomized controlled prospective study." Acta ophthalmologica **95**(7): e610-e618.

Purpose: To evaluate the efficacy and safety of eplerenone for chronic nonresolving central serous chorioretinopathy (CSC). Methods: Prospective, double-blind, randomized placebo-controlled study. Nineteen eyes of 17 patients with persistent subretinal fluid (SRF) due to CSC were enrolled and randomized to receive eplerenone 50 mg/day or placebo for 3 months, followed by a 3-month follow-up. The main outcome measure was change in SRF from baseline to 3 months of treatment. Secondary outcomes included change in SRF at any time-point, complete resolution of SRF, improvement in choroidal thickness and change in best-corrected visual acuity (BCVA). Results: Thirteen eyes were treated with eplerenone and six with placebo. Both groups showed reduction in SRF throughout the treatment period, with a significant reduction at months 1, 3 and 5 only in the treatment group. Twenty-three per cent in the treatment group and 30.8% per cent in the placebo group experienced complete resolution of SRF. A significant improvement in BCVA was noted in the placebo group at 4 months, as well as a significant difference in BCVA between groups at 3 months in favour of the placebo group (p = 0.005). There was no significant difference in choroidal thickness in either group throughout the study period. No adverse events related to eplerenone were noted in the treatment group. Conclusion: In this study, eplerenone was not found to be superior to placebo in eyes with chronic CSC. Copyright © 2017 Acta Ophthalmologica Scandinavica Foundation. Published by John Wiley & Sons Ltd

Semeraro, F., et al. (2012). "Intravitreal bevacizumab versus low-fluence photodynamic therapy for treatment of chronic central serous chorioretinopathy." Japanese journal of ophthalmology **56**(6): 608-612.

PURPOSE: To report the effectiveness and safety of intravitreal bevacizumab injection (IVB) compared with low-fluence photodynamic therapy (L-PDT) in eyes with chronic central serous chorioretinopathy (CSC). METHODS: This was a prospective comparative interventional study of 22 patients affected by chronic CSC. Patients were randomly assigned to group 1 (12 patients

treated with 1.25 mg IVB) and group 2 (10 patients treated with L-PDT). In group 2, PDT with verteporfin was delivered at low fluence (300 mW/cm(2) for 83 s, 25 J/cm(2)). Follow-up visits were scheduled at 1, 3, 6, and 9 months. RESULTS: The improvement in visual acuity was greater in group 1 than in group 2, although the difference was not statistically significant (P = 0.59). The mean change in central point thickness over 9 months from baseline was 127 µm (SD 36) in group 1 and 114 µm (SD 42) in group 2. After the first injection, retreatments were given if recurrence was noted, with an average of 3.0 ± 1 injections in group 1 and 1.6 ± 0.6 treatments with L-PDT in group 2. The difference between retreatment in the 2 groups was not statistically significant (P = 0.45). CONCLUSIONS: Based on the results obtained after 9 months of follow-up, our study provides evidence that IVB may be a treatment option for chronic CSC.

Shao, Y., et al. (2017). "Clinical findings associated with propranolol and fenofibrate on acute central serous chorioretinopathy." International journal of clinical and experimental medicine **10**(1): 638-647.

Objective: To evaluate the effects of fenofibrate with propranolol in the pathophysiology of acute central serous chorioretinopathy (CSCR). Methods: Totally 48 patients (48 eyes) with a history of acute CSCR were randomed divided into two groups: group A was treated with a combination of fenofibrate (200 mg) and propranolol (60 mg, 3 times daily) for 8 weeks, and group B with only fenofibrate (200 mg, once daily). The visual acuity, subjective symptom, OSDI, tear film test with 4 terms, tear protein and optical coherence tomography [including mean central subfield thickness (CST), mean subretinal fluid volumn (SFV), mean subretinal fluid vertical diameter (SFVD) and mean subretinal fluid horizontal diameter (SFHD)] were observed at every other week before and after treatment. Results: After treatment, the average baseline BCVA was 0.35 logMAR for group A and 0.36 logMAR for group B, respectively. And the average BCVA was 0.21 logMAR and 0.27 logMAR, for each group respectively. The differences of improved BCVA before and after treatment between the two groups were statistically significant (P<0.05). In group A, the average CST was 181.54 µm and baseline SFV was 0.17 µm. The average SFVD was 28.63 µm and SFHD was 203.83 µm. The decrease of CST, SFV, SFVD and SFHD was statistically significant at the fourth follow-up (6-8 weeks after treatment) compared with baseline (P=0.021, 0.018, 0.029, and 0.017, respectively). In group B, the average CST was 226.88 µm and baseline SFV was 0.40 µm. The average SFVD was 56.54 µm and SFHD was 654.54 µm. These parameters were also statistically significantly decreased at the fourth follow-up period (6-8 weeks after treatment) compared with baseline (P=0.041, 0.025, 0.033, and 0.011, respectively). We also observed significant differences between these two groups for the CST, SFV, SFVD and SFHD at the fourth follow-up (all P<0.05). Conclusion: Fenofibrate combined with propranolol is more clinically efficient than fenofibrate only in the treatment of patients with CSCR.

Shinojima, A., et al. (2017). "A multicenter randomized controlled study of antioxidant supplementation with lutein for chronic central serous chorioretinopathy." Ophthalmologica **237(3)**: 159-166.

Purpose: To investigate functional and morphological changes in patients with chronic central serous chorioretinopathy after supplementation with antioxidants containing lutein or a placebo. Procedures: One hundred eyes of 100 patients were randomly divided into 2 groups, one taking tablets with lutein plus other antioxidants and the other taking a placebo for 6 months. Best-corrected visual acuity (BCVA) and the subfoveal fluid height on optical coherence tomography were measured. Result(s): Seventy-nine patients (37 in the supplementation and 42 in the placebo group) completed the 6-month follow-up. In the supplementation group, mean BCVA showed significant improvement (p = 0.003), while there was no significant change in the placebo group (p = 0.589). The mean subfoveal fluid height was significantly reduced, by 28.6%, in the supplementation group (p = 0.028), in contrast to 3.3% in the placebo group (p = 0.898).

Conclusion(s): Antioxidant supplementation significantly reduced subfoveal fluid height. The impacts of antioxidant supplementation on BCVA remain to be elucidated in future studies. Copyright © 2017 S. Karger AG, Basel.

Staurenghi, G., et al. (2018). "Efficacy and Safety of Ranibizumab 0.5 mg for the Treatment of Macular Edema Resulting from Uncommon Causes." Ophthalmology **125**(6): 850-862.

Purpose: To evaluate the efficacy and safety of ranibizumab 0.5 mg in adult patients with macular edema (ME) resulting from any cause other than diabetes, retinal vein occlusion, or neovascular age-related macular degeneration. Design: A phase 3, 12-month, double-masked, randomized, sham-controlled, multicenter study. Participants: One hundred seventy-eight eligible patients aged >= 18 years. Methods: Patients were randomized 2: 1 to receive either ranibizumab 0.5 mg (n = 118) or sham (n = 60) at baseline and month 1. From month 2, patients in both arms received open-label individualized ranibizumab treatment based on disease activity. A preplanned subgroup analysis was conducted on the primary end point on 5 predefined baseline ME etiologies (inflammatory/post-uveitis, pseudophakic or aphakic, central serous chorioretinopathy, idiopathic, and miscellaneous). Main Outcome Measures: Changes in best-corrected visual acuity (BCVA; Early Treatment Diabetic Retinopathy Study letters) from baseline to month 2 (primary end point) and month 12 and safety over 12 months. Results: Overall, 156 patients (87.6%) completed the study. The baseline characteristics were well balanced between the treatment arms. Overall, ranibizumab showed superior efficacy versus sham from baseline to month 2 (least squares mean BCVA, +5.7 letters vs. +2.9 letters; 1-sided P = 0.0111), that is, a treatment effect (TE) of +2.8 letters. The mean BCVA gain from baseline to month 12 was 9.6 letters with ranibizumab. The TE at month 2 was variable in the 5 predefined etiology subgroups, ranging from > 5-letter gain to 0.5-letter loss. The safety findings were consistent with the well-established safety profile of ranibizumab. Conclusions: The primary end point was met and ranibizumab showed superiority in BCVA gain over sham in treating ME due to uncommon causes, with a TE of +2.8 letters versus sham at month 2. At month 12, the mean BCVA gain was high (9.6 letters) in the ranibizumab arm; however, the TE was observed to be variable across the different etiology subgroups, reaching a > 1-line TE in BCVA in patients with ME resulting from inflammatory conditions/post-uveitis or after cataract surgery. Overall, ranibizumab was well tolerated with no new safety findings up to month 12. (C) 2018 by the American Academy of Ophthalmology.

Subhi, Y., et al. (2022). "Subretinal fluid morphology in chronic central serous chorioretinopathy and its relationship to treatment: a retrospective analysis on PLACE trial data." Acta ophthalmologica **100**(1): 89-95.

Purpose: To explore subretinal fluid (SRF) morphology in chronic central serous chorioretinopathy (cCSC) after one session of either high-density subthreshold micropulse laser (HSML) treatment or half-dose photodynamic therapy (PDT). Methods: We retrospectively obtained optical coherence tomography (OCT) scans from a subset of patients from a randomized controlled trial on treatment-naïve eyes with cCSC allocated to either HSML treatment or half-dose PDT. OCT scans were evaluated prior to treatment and 6–8 weeks post-treatment, where we measured maximum SRF height and width, calculated the maximum height-to-maximum width-ratio (maxHWR) and calculated the total SRF volume. Results: Forty-one eyes of 39 cCSC patients were included. SRF morphology ranged from flat to dome-shaped, quantified as maxHWR ranging between 0.02 and 0.12. SRF volume was median 0.373 μl (range: 0.010–4.425 μl) and did not correlate to maxHWR (rho = −0.004, p = 0.982). Half-dose PDT was superior to HSML treatment in complete SRF resolution (RR = 3.28, p = 0.003) and in morphological changes of SRF (Δmaximum height, p = 0.001; Δmaximum width, p < 0.001; Δvolume, p = 0.025). SRF resolved completely in 19/22 PDT-

treated eyes (86%) and 5/19 HSML-treated eyes (26%). SRF volume increased in five eyes (26%) after HSML treatment, and in none of the eyes after half-dose PDT. SRF morphology at baseline did not predict treatment outcomes. Conclusion: SRF morphology changed after both HSML treatment and half-dose PDT in cCSC, with SRF disappearing in most PDT-treated patients, whereas SRF volume increased in a sizeable proportion of HSML-treated patients. Baseline SRF characteristics measured in this study were unable to predict outcomes after either HSML treatment or half-dose PDT.

Sun, X., et al. (2018). "Spironolactone versus observation in the treatment of acute central serous chorioretinopathy." British journal of ophthalmology **102**(8): 1060-1065.

PURPOSE: To evaluate the efficacy of oral spironolactone in patients with acute central serous chorioretinopathy (CSC). METHODS: This is a prospective, randomised controlled clinical study. Thirty patients with acute CSC were the participants, including 18 patients who were treated with spironolactone (40 mg orally, twice daily) for 2 months in the experimental group and 12 patients who received observation in the control group. Main outcome measures included the proportion of eyes achieving complete resolution of subretinal fluid (SRF), changes in central macular thickness (CMT), the height of SRF (SRFH), best corrected visual acuity (BCVA) and subfoveal choroidal thickness (SFCT). The follow-up period was 2 months. RESULTS: Complete resolution of SRF was achieved in 55.6% (10/18) and 8.3% (1/12) of the eyes in the treatment group and the control group, respectively, at 2 months (p=0.018). The mean CMT and SRFH decreased significantly at each visit in both groups (p<0.05), and there was significant difference between the two groups at 2 months (p<0.05 and p<0.05, respectively). BCVA (in logarithm of the minimum angle of resolution; mean) improved in both groups at 2 months (p<0.05). In the treatment group, the mean baseline SFCT significantly decreased from 502.50±87.38 μm to 427.44±74.37 μm at 2 months (p<0.01), while the change from baseline (from 480.33±102.38 μm to 463.75±100.63 μm) was not significant in the control group (p=0.195). But the differences between the two groups in BCVA and SFCT were not significant. CONCLUSIONS: Oral spironolactone is more effective with a faster absorption of SRF than observations. It is a promising treatment for acute CSC. TRIAL REGISTRATION NUMBER: ChiCTR-IPR-16008428, Results.

Sun, Z. H., et al. (2020). "Efficacy and safety of subthreshold micropulse laser compared with threshold conventional laser in central serous chorioretinopathy." Eye **34**(9): 1592-1599.

Purpose To compare the efficacy and safety of subthreshold micropulse laser (SML) with threshold conventional laser (TCL) in central serous chorioretinopathy (CSC). Methods Prospective, randomized, double-masked, non-inferiority, 12-week clinical trial. Patients were randomly assigned 1:1 to SML group or TCL group. Patients in the SML group were treated with 577 nm micropulse laser. The spot size was 160 mu m, the duty cycle was 5% and exposure time was 0.2 s. The power was 50% threshold tested. Patients in the TCL group were treated with 577 nm continuous laser. The power was 100% threshold tested. The primary outcome was the mean change in best-corrected visual acuity (BCVA) at week 12, with a non-inferiority limit of five letters on the Early Treatment Diabetic Retinopathy Study (ETDRS) visual acuity charts. Results Eighty-eight patients were enroled. Seventy-seven patients were male. Forty-four patients were in SML group and 44 in TCL group. At week 12, SML was equivalent to TCL with a gain of 6.23 +/- 8.59 and 6.61 +/- 6.35 letters, respectively, (SML-TCL difference: -0.38 letters; 95% confidence interval (CI):-3.58-2.81;Pnon-inferiority = 0.0026). There was no statistically significant difference between the two groups (t = 0.240,P = 0.811). At week 12, the proportion of patients whose SRF had been totally absorbed was 63.63 and 81.82% respectively for SML and TCL groups. There was no statistically significant difference between the two groups (chi(2) = 3.67,P = 0.056). Conclusions

Both SML and TCL can improve visual acuity in CSC. SML was non-inferior to TCL in the improvement of BCVA.

Tvenning, A. O., et al. (2019). "Treatment of large avascular retinal pigment epithelium detachments in age-related macular degeneration with aflibercept, photodynamic therapy, and triamcinolone acetonide." Clinical ophthalmology (Auckland, N.Z.) **13**: 233-241.

Purpose: To evaluate the use of aflibercept, triamcinolone acetonide, and photodynamic therapy (PDT) in the treatment of avascular pigment epithelium detachments (aPEDs). Patients and methods: Patients with treatment-naïve aPEDs ≥1,500 μm in diameter were randomized to treatment or observation. Treatment consisted of 6 monthly intravitreal injections of aflibercept. If the aPED persisted, the patients were treated with half-fluence PDT in combination with intravitreal triamcinolone acetonide and aflibercept. The primary outcome was change of best-corrected visual acuity (BCVA) after 24 months of follow-up. Secondary outcomes were changes in pigment epithelium volume, height and diameter, central retinal thickness, and number of patients developing choroidal neovascularization or geographic atrophy (GA). Results: Treatment and inclusion of patients were stopped after an interim analysis of 6-month data because 75% of the aPEDs were in different stages of GA. Nine patients with aPED were included in the study, of these one patient was excluded because of bilateral central serous chorioretinopathy. The remaining eight had drusenoid aPEDs. After 24 months of follow-up, estimated means of BCVA decreased by 4.2 and 20.8 letters in the treatment and observation group, respectively. This decrease over time was not significantly different between groups (P=0.140, 95% CI-5.3, 38.6). Estimated means of PED volume, height, diameter, and central retinal thickness were not significantly different between groups. Choroidal neovascularization and retinal pigment epithelium tear developed in one patient in the treatment group. One patient in the treatment group and two patients in the observation group progressed to complete retinal pigment epithelium and outer retinal atrophy. A decrease in PED volume was associated with the development of complete retinal pigment epithelium and outer retinal atrophy (P=0.029). Conclusion: This small trial indicates that multitargeted, primarily antiangiogenic therapy does not favorably alter the natural course of drusenoid aPEDs.

van Dijk, E. H. C., et al. (2018). "Half-Dose Photodynamic Therapy versus High-Density Subthreshold Micropulse Laser Treatment in Patients with Chronic Central Serous Chorioretinopathy: The PLACE Trial." Ophthalmology **125(10)**: 1547-1555.

Purpose: To compare the anatomic and functional efficacy and safety of half-dose photodynamic therapy (PDT) versus high-density subthreshold micropulse laser (HSML) treatment in patients with chronic central serous chorioretinopathy (cCSC). Design(s): Open-label, multicenter, randomized controlled clinical trial. Participant(s): Patients with cCSC whose disease had to be confirmed by both clinical characteristics and findings on multimodal imaging. Method(s): Eligible patients were randomized in a 1:1 allocation ratio. Treatment was evaluated during a follow-up visit, and the same treatment was repeated in patients who still demonstrated subretinal fluid (SRF). Main Outcome Measure(s): The primary end point was the complete disappearance of SRF at the first evaluation visit at 6 to 8 weeks after treatment. As a secondary outcome measure, we assessed this anatomic result at the final evaluation visit at 7 to 8 months after treatment. Other secondary outcomes covered functional improvement and included change in best-corrected visual acuity (BCVA; measured in Early Treatment Diabetic Retinopathy Study [ETDRS] letters), retinal sensitivity (measured using microperimetry), and vision-related quality of life using a validated questionnaire. Result(s): Between November 2013 and September 2016, 179 patients were included: 89 patients were assigned randomly to half-dose PDT, and 90 were assigned randomly to HSML treatment. At their first evaluation visit, SRF had resolved in 51.2% and 13.8% of patients,

respectively (P < 0.001). At their final evaluation visit, a significantly higher percentage of PDT-treated patients demonstrated no SRF (67.2% vs. 28.8%; P < 0.001). Moreover, at the first evaluation visit, the PDT-treated patients showed a significantly higher increase in BCVA (+4.60+/-6.62 ETDRS letters vs. +1.39+/-8.99 ETDRS letters; P = 0.011), and a significantly higher increase in retinal sensitivity on microperimetry (+2.01+/-3.04 dB vs. +0.92+/-3.65 dB; P = 0.046); however, the improvement in vision-related quality of life was similar (score of +2.87+/-8.35 vs. +2.56+/-7.36, respectively; P = 0.800). Conclusion(s): Half-dose PDT is superior to HSML for treating cCSC, leading to a significantly higher proportion of patients with complete resolution of SRF and functional improvement. Copyright © 2018 American Academy of Ophthalmology

Van Rijssen, T., et al. (2022). "Twenty-four months follow-up of chronic central serous chorioretinopathy patients after treatment with oral eplerenone or half-dose photodynamic therapy, and crossover treatment: SPECTRA trial report No. 4." Acta ophthalmologica **100**: 18.

Purpose: To compare the efficacy and safety of primary treatment with either half-dose photodynamic therapy (PDT) or oral eplerenone, or crossover treatment in patients with chronic central serous chorioretinopathy. Methods: After baseline visit of the SPECTRA trial, patients were randomized to either half-dose PDT or oral eplerenone treatment. Patients with persistent subretinal fluid (SRF) on optical coherence tomography (OCT) at first follow-up (at 3 months) visit received crossover treatment. Presence of SRF, best-corrected visual acuity (BCVA), and retinal sensitivity on microperimetry were evaluated at 24 months after baseline visit. Results: There were 80 patients who could be evaluated at 24 months. In the patients primarily randomized to half-dose PDT, complete SRF resolution was present in 32/40 (80.0%) patients (including 8 patients who received crossover treatment with eplerenone) at 24 months, while this was the case in 35/40 (87.5%) of patients who were primarily randomized to eplerenone (including 32 patients who received crossover treatment with half-dose PDT). There were no significant differences in best-corrected visual acuity, retinal sensitivity or visual functioning questionnaire score. Conclusions: A large portion of patients treated with half-dose PDT (either as primary or crossover treatment) still had a complete SRF resolution 24 months after baseline. A delay of 3 months for half-dose PDT after baseline does not appear to have significant negative consequences in the long term.

van Rijssen, T. J., et al. (2021). "RESPONSE OF CHOROIDAL ABNORMALITIES TO PHOTODYNAMIC THERAPY VERSUS MICROPULSE LASER IN CHRONIC CENTRAL SEROUS CHORIORETINOPATHY: place Trial Report No. 4." Retina (Philadelphia, Pa.) **41**(10): 2122-2131.

PURPOSE: To compare the effects of half-dose photodynamic therapy (PDT) and high-density subthreshold micropulse laser on choroidal dysfunction evaluated by degree and extent of hyperfluorescence on indocyanine green angiography (ICGA) in chronic central serous chorioretinopathy. METHODS: Data from the multicenter, randomized, controlled PLACE trial were used in this study. Hyperfluorescent and hypofluorescent areas on ICGA, their association with subretinal fluid and visual function were assessed. RESULTS: In total, 146 patients were included (72 in the PDT and 74 in the high-density subthreshold micropulse laser treatment arm). A significantly greater decrease in the size of hyperfluorescent areas on ICGA at first visit after treatment was seen after PDT compared with high-density subthreshold micropulse laser (mean, -1.41 ? 2.40 mm2 vs. -0.04 ? 0.73 mm2, respectively; P < 0.001). A reduction in the degree of hyperfluorescence on ICGA decreased the odds of having persistent subretinal fluid on optical coherence tomography at first visit after treatment (B = 0.295; P = 0.019). There were no significant differences in best-corrected visual acuity and retinal sensitivity between the subgroup with novel hypofluorescence (n = 20, 28%) on ICGA at first visit post PDT, compared with the subgroup without novel hypofluorescence on ICGA after PDT. CONCLUSION: Choroidal abnormalities in

chronic central serous chorioretinopathy can be effectively treated by ICGA-guided half-dose PDT but not with high-density subthreshold micropulse laser application.

van Rijssen, T. J., et al. (2020). "Prospective evaluation of changes in choroidal vascularity index after half-dose photodynamic therapy versus micropulse laser treatment in chronic central serous chorioretinopathy." Graefe's archive for clinical and experimental ophthalmology **258(6)**: 1191-1197.

Purpose: To assess whether treatment of chronic central serous chorioretinopathy (cCSC) with photodynamic therapy (PDT) and high-density subthreshold micropulse laser (HSML) results in choroidal vascularity index (CVI) changes that may account for the treatment effect. Method(s): Patients with cCSC were prospectively included and analyzed. Patients received either half-dose PDT or HSML treatment. CVI of the affected and unaffected eye was obtained before treatment, 6 to 8 weeks after treatment, and 7 to 8 months after treatment. Result(s): At baseline, 29 eyes (29 patients) were included both in the PDT and in the HSML group. The mean (+/- standard deviation) CVI change in the HSML group between before PDT and 6 to 8 weeks after PDT was - 0.009 +/- 0.032 (p = 0.127), whereas this was 0.0025 +/- 0.037 (p = 0.723) between the visit before PDT and final visit. The patients in the PDT group had a CVI change of - 0.0025 +/- 0.037 (p = 0.723) between the visit before PDT and first visit after PDT, and a mean CVI change of - 0.013 +/- 0.038 (p = 0.080) between the visit before PDT and final visit. There was no significant correlation between CVI and BCVA at the measured time points, in both the HSML group (p = 0.885), and in the PDT group (p = 0.904). Moreover, no significant changes in CVI occurred in the unaffected eye at any time point. Conclusion(s): PDT and HSML do not significantly affect CVI, and therefore a CVI change may not be primarily responsible for the treatment effect. The positive treatment effect of both interventions may rely on other mechanisms, such as an effect on choriocapillaris and/or retinal pigment epithelium function. Copyright © 2020, The Author(s).

van Rijssen, T. J., et al. (2019). "Focal and Diffuse Chronic Central Serous Chorioretinopathy Treated With Half-Dose Photodynamic Therapy or Subthreshold Micropulse Laser: PLACE Trial Report No. 3." American journal of ophthalmology **205**: 1-10.

PURPOSE: To compare the outcome between high-density subthreshold micropulse laser (HSML) treatment and half-dose photodynamic therapy (PDT) in chronic central serous chorioretinopathy (cCSC) patients, subdivided based on either focal or diffuse leakage on fluorescein angiography (FA). DESIGN: Retrospective analysis of multicenter randomized controlled trial data. METHODS: Patients were treated with either half-dose PDT or HSML (both indocyanine green angiography-guided) and categorized in 2 groups, based on focal or diffuse leakage on FA. Clinical outcomes were evaluated at baseline and during follow-up. RESULTS: In the focal leakage group (63 patients), both at first evaluation and at final visit, more PDT-treated than HSML-treated patients demonstrated a resolution of subretinal fluid (evaluation visit 1: 57% in the PDT group and 17% in the HSML group, P = .007; final visit: 75% and 38%, P = .012). In the diffuse leakage group (93 patients), both at first evaluation and at final visit, more PDT-treated than HSML-treated patients showed a resolution of subretinal fluid (evaluation visit: 1:48% in the PDT group and 16% in the HSML group, P = .002; final visit: 67% and 21%, P = .002). PDT-treated patients in the focal and diffuse leakage group had a higher retinal sensitivity increase, comparing baseline and final visit (+3.1 ± 3.1 dB vs +1.2 ± 4.0 dB, P = .048, and +2.7 ± 3.3 dB vs +1.0 ± 3.8 dB, P = .036, respectively). Only in the diffuse leakage group, the increase in ETDRS letters was higher in the PDT-treated group when comparing baseline and first evaluation visit (+4.4 ± 6.1 vs +0.9 ± 10.0, P = .049). CONCLUSIONS: Half-dose PDT is superior to HSML treatment in cCSC patients, regardless of the presence of focal or diffuse leakage on FA.

van Rijssen, T. J., et al. (2020). "Crossover to Photodynamic Therapy or Micropulse Laser After Failure of Primary Treatment of Chronic Central Serous Chorioretinopathy: The REPLACE Trial." Am J Ophthalmol **216**: 80-89.

PURPOSE: To assess whether chronic central serous chorioretinopathy (cCSC) patients without a complete resolution of subretinal fluid (SRF) after either half-dose photodynamic therapy (PDT) or high-density subthreshold micropulse laser (HSML) treatment may benefit from crossover treatment. DESIGN: Multicenter prospective interventional case series. METHODS: cCSC patients with persistent SRF at the final visit of the PLACE trial were included. Patients received crossover treatment with either half-dose PDT or HSML. RESULTS: Thirty-two patients received PDT and 10 patients received HSML. At the first evaluation visit (6-8 weeks after treatment), 81% of patients in the PDT group had complete resolution of SRF, while none of the HSML-treated patients had complete resolution of SRF. At final visit (1 year after baseline), 78% (P = .030) and 67% (P = .109) of the patients, respectively, had a complete resolution of SRF. The mean retinal sensitivity in the PDT group increased from 21.7 dB (standard error [SE]: 0.9) to 23.4 dB (SE: 0.8) at evaluation visit 1 (P = .003), to 24.7dB (SE: 0.8) at final visit (P < .001), while there were no significant changes in the HSML group (23.7 dB [SE: 1.6] at baseline, 23.8 dB [SE: 1.4] at evaluation 1, and 23.3 dB [SE: 1.4] at final visit). The mean visual acuity and mean visual quality-of-life questionnaire score did not change significantly in both groups. CONCLUSIONS: Crossover to half-dose PDT after previous unsuccessful HSML treatment for cCSC may lead to improved anatomic and functional endpoints, while crossover to HSML after half-dose PDT does not seem to significantly affect these endpoints.

van Rijssen, T. J., et al. (2022). "Half-Dose Photodynamic Therapy Versus Eplerenone in Chronic Central Serous Chorioretinopathy (SPECTRA): a Randomized Controlled Trial." American journal of ophthalmology **233**: 101-110.

PURPOSE: To compare the efficacy and safety between half-dose photodynamic therapy (PDT) and eplerenone therapy for treating chronic central serous chorioretinopathy (cCSC). DESIGN: This was a multicenter, open-label, randomized controlled trial. METHODS: This investigator-initiated trial was conducted in 3 academic medical centers in the Netherlands. Eligible patients were randomized at a 1:1 ratio to receive either indocyanine green angiography-guided half-dose PDT or oral eplerenone for 12 weeks. Both anatomical and functional outcomes were evaluated at 3 months after the start of treatment. RESULTS: A total of 107 patients were randomly assigned to receive either half-dose PDT (n = 53) or eplerenone treatment (n = 54). Thirteen patients (3 in the PDT group and 10 in the eplerenone group) did not adhere to the study protocol. At the 3-month evaluation visit, 78% of patients in the PDT group had complete resolution of subretinal fluid accumulation compared to only 17% of patients in the eplerenone group (P < .001). Mean best-corrected visual acuity in Early Treatment of Diabetic Retinopathy Study letters at the 3-month evaluation visit was 83.7 ± 10.8 and 82.8 ± 9.0 in the PDT and eplerenone groups, respectively (P = .555). In addition, mean retinal sensitivity on microperimetry was 25.4 ± 3.4 dB and 23.9 ± 4.0 dB in the PDT and eplerenone groups, respectively (P = .041). Finally, mean vision-related quality of life scores were 87.2 ± 8.5 and 83.8 ± 12.1 in the PDT and eplerenone groups, respectively (P = .094). Three patients (6%) in the PDT group experienced adverse events during the study compared to 18 patients (33%) in the eplerenone group. CONCLUSIONS: Half-dose PDT is superior to oral eplerenone for cCSC with respect to both short-term safety and efficacy outcomes.

Venkatesh, R., et al. (2020). "Oral eplerenone versus observation in the management of acute central serous chorioretinopathy: a prospective, randomized comparative study." Pharmaceuticals (Basel, Switzerland) **13**(8): 1-11.

In this prospective, interventional case-control study, 58 patients with unilateral acute central serous chorioretinopathy (CSCR) were recruited. Patients ≥ 18 years age, presenting with first episodes of acute CSCR, were included. Acute CSCR was defined by the presence of subretinal fluid (SRF) and symptoms for <12 weeks duration with no clinical or imaging features of chronicity. Patients were alternately divided into treatment (Table Eplerenone 50 mg/day for minimum 1 month) and observation groups. Vision, SRF height and subfoveal choroidal thickness (SFCT) were checked at 1-, 2-and 3-months in both eyes of each group. Each group had 29 eyes. Mean age was 40.4 ± 7.1 and 43.3 ± 8.34 years in treatment and observation group, respectively. Mean symptom duration was 6.46 ± 1.45 and 5.87 ± 2.09 weeks, respectively. Vision improvement to 6/6 was seen in 92%, 100% and 100% cases in treatment group and 74%, 86% and 100% in control group at each visit, respectively. Complete SRF resolution in the treatment group was noted in 45%, 55% and 62% cases at each respective monthly visit. In the observation group, complete SRF resolution was noted in 10%, 21% and 31% at 1-, 2-and 3-month visits, respectively. SRF ($p < 0.001$) and SFCT ($p < 0.001$) reduction was noted in the affected eye of both groups. SFCT was reduced in the fellow eye after treatment ($p = 0.005$) compared to the observation group ($p = 0.276$). In conclusion, oral eplerenone achieves faster SRF resolution and vision improvement in acute CSCR. Additionally, it shows beneficial effects on the fellow eye.

Wang, T. T. and G. X. Xu (2009). "Clinical observation of alliance application of compound anisodine and joletion in the treatment of central serous chorioretinopathy." International journal of ophthalmology **9**(6): 1169-1171.

• AIM: To investigate the effects of compound anisodine combined with joletion tablets in the treatment of central serous chorioretinopathy (CSC). • METHODS: A random control study was performed in which the 57 cases CSC cases were assigned randomly into 2 groups: 26 cases (control group) received only joletion tablets three times one day, 1.5mg each time; 31 cases (therapeutic alliance group) were injected subcutaneously compound anisodine injection 2mL q. d around superficial temporal arteries in the affected eyes and combined with joletion tablets taking. Both groups received therapy for 30 days. Before therapy and 2 weeks and 10 weeks after therapy each case was examined with: subjective symptom, the best corrected visual acuity, optical coherent topography (OCT) of fundus and average light sensitivity. • RESULTS: When all the patients were examined 2 weeks after therapy, the differences of the recoveries of subjective symptom, the best corrected visual acuity, OCT of fundus and average light sensitivity between the 2 groups were statistically significant ($P < 0.05$). When all the patient were examined 10 weeks after therapy, the differences of the recoveries of subjective symptom,the best corrected visual acuity and OCT of fundus between the 2 groups were not statistically insignificant ($P > 0.05$), but average light sensitivity was on the contrary ($P<0.05$). Of all the patients, no severe adverse effect was found during the therapy. • CONCLUSION: Compound anisodine combined with joletion tablets may be a kind of safe and effective therapy for CSC for it can shorten the course of CSC and protect the visual function of macula lutea.

Wei, R., et al. (2016). "Effect of fenofibrate and aspirin on acute central serous chorioretinopathy." International journal of clinical and experimental medicine **9**(8): 15996-16005.

Background: Central serous chorioretinopathy (CSCR) may result in serous elevation of the retinal pigment epithelium (RPE) and/or detachment of the neural retina due to RPE barrier dysfunction. Fenofibrate acts as an efficacy medicine could improve the vision and symptoms of patients in patients with CSCR. In this study, we continue to define the effectiveness of this new treatment strategy by comparing the results obtained in patients treated with fenofibrate and aspirin with those in a historic control group consisting of patients with CSCR who were only taking fenofibrate.

Methods: Totally 60 patients (60 eyes) with a history of acute CSCR on fenofibrate were randomized into two groups: A combination of fenofibrate (200 mg) and aspirin (100 mg) was used in group A, whereas in group B, only fenofibrate (200 mg) was administered. They were taken before meals half an hour and 1 times per day for 8 weeks. The change of the best corrected visual acuity (BCVA) and coherence tomography [including mean central subfield thickness (CST), mean subretinal fluid volumn (SFV), mean subretinal fluid vertical diameter (SFVD), mean subretinal fluid horizontal diameter (SFHD)] were observed at 1, 2, 4, 8 weeks before and after treatment. Results: After treatment, the average baseline BCVA (logMAR) was 0.34 and the average BCVA (logMAR) was 0.23 in Group A. In group B, the average baseline BCVA (logMAR) was 0.35 and the average BCVA (logMAR) was 0.28 at study completion. The differences of improved BCVA before and after treatment between the two groups were statistically significant (P<0.05). Besides, the CST, SFV, SFVD and SFHD significantly decreased 49.5%, 78.8%, 79.3%, 90.5% and had statistically significant at the fourth follow-up compared with baseline (= 0.031, = 0.014, = 0.022, and = 0.019, respectively) in group A. The CST, SFV, SFVD and SFHD significantly decreased 37.0%, 57.2%, 58.8%, 73.0% and had statistically significant at the fourth follow-up compared with baseline (= 0.046, = 0.036, = 0.049, and = 0.057, respectively). There were significant difference for the CST, SFV, SFVD and SFHD at the fourth follow-up in both groups (all P<0.05). Conclusion: Fenofibrate has more clinical efficacy in the treatment of patients with CSCR combined with asprin than fenofibrate only.

Wu, Z., et al. (2011). "Improvement in multifocal electroretinography after half-dose verteporfin photodynamic therapy for central serous chorioretinopathy: A randomized placebo-controlled trial. []." Retina. **03**.

PURPOSE: To evaluate retinal functional changes by multifocal electroretinography (mfERG) after photodynamic therapy with half-dose verteporfin in patients with acute central serous chorioretinopathy. METHOD(S): Thirty-four patients with acute central serous chorioretinopathy were randomly assigned to receive photodynamic therapy with half-dose verteporfin (n = 24) or placebo (n = 10). Multifocal electroretinography was performed at baseline and at 12 months, and serial changes in response amplitudes were expressed as amplitude ratios. The mfERG amplitude ratios, best-corrected visual acuity, and optical coherence tomography central foveal thickness were compared between the verteporfin and placebo groups. Correlation analysis between the mfERG response amplitude ratios and the best-corrected visual acuity changes and reduction in optical coherence tomography central foveal thickness were also performed. RESULT(S): At 12 months, the mean visual improvement was 1.8 line and 0.1 line for the verteporfin and placebo groups, respectively (P = 0.003). Eyes in the verteporfin group had significantly lower central foveal thickness (P = 0.028) and higher P1 mfERG response ratios for Rings 1 and 2 at 12 months compared with the eyes in the placebo group (P = 0.030 and P = 0.018, respectively). Significant correlations between mfERG N1 and P1 amplitude ratios at the central rings were observed with both changes in best-corrected visual acuity and reductions in optical coherence tomography central foveal thickness (P < 0.05). CONCLUSION(S): Multifocal electroretinography demonstrated higher retinal function at the central macula objectively in central serous chorioretinopathy patients treated with half-dose verteporfin photodynamic therapy. Changes in best-corrected visual acuity and optical coherence tomography central foveal thickness findings also correlated with mfERG responses of the central macula, confirming the usefulness of mfERG as an objective investigation to evaluate the functional changes in central serous chorioretinopathy.

Xu, J. F. and K. C. Chen (2014). "Treatment of juxtafoveal central serous chorioretinopathy by compound anisodine injection." International eye science **14**(4): 701-703.

AIM: To investigate the efficiency and security of compound anisodine injection in the treatment of juxtafoveal central serous chorioretinopathy (CSC). METHODS: Sixty patients (60 eyes) who were diagnosed of juxtafoveal CSC were assigned randomly into 2 groups: 32 cases (32 eyes, therapeutic group) were injected subcutaneously compound anisodine injection for 2 mL q. d around superficial temporal arteries in the affected eyes; 28 cases (28 eyes, control group) received only traditional oral medication. Both groups received therapy for 2 to 4 courses of treatment. The main observations were the best corrected visual acuity (BCVA), subjective symptom, visual field, average light sensitivity and optical coherent topography (OCT). RESULTS: There was no significant difference between the therapeutic group and the control group before treatment (P>0.05), but all the outcome measures at 1, 3mo in the treatment group were significantly improved as compared with control group (P<0.05). After 6 mo, there were no significant difference between the two groups in all measures (P>0.05). No severe adverse reaction was noted except mild ones such as temporary dry mouth, dizziness and palpitation in a few cases. CONCLUSION: Compound anisodine injection has remarkable effects in the treatment of juxtafoveal CSC. It can shorten the course, improved the visual function and decreased the recurrence rate of CSC.

Zhao, M. W., et al. (2015). "A 50% vs 30% Dose of Verteporfin (Photodynamic Therapy) for Acute Central Serous Chorioretinopathy One-Year Results of a Randomized Clinical Trial." JAMA ophthalmology **133**(3): 333-340.

IMPORTANCE A randomized clinical trial is needed to evaluate what is the best photodynamic therapy (PDT) protocol to use for acute central serous chorioretinopathy. OBJECTIVE To compare the efficacy and safety of a 50% dose of verteporfin (a method of PDT) with the efficacy and safety of a 30% dose for acute central serous chorioretinopathy. DESIGN, SETTING, AND PARTICIPANTS A multicenter, noninferiority, double-masked, randomized, controlled, clinical trial in which 131 patients (131 eyes) with acute central serous chorioretinopathy for less than 6 months were recruited with a follow-up of 12 months from university-based ophthalmology practices. INTERVENTIONS Patients were randomly assigned to either a 50% dose of verteporfin (the 50%-dose PDT group) or a 30% dose (the 30%-dose PDT group). MAIN OUTCOMES AND MEASURES The 2 primary outcome measures were the proportion of eyes with complete absorption of subretinal fluid and the proportion of eyes with complete disappearance of fluorescein leakage at 6 and 12 months. The secondary outcome measures included the subretinal fluid recurrent rate, the fluorescein leakage recurrent rate at 12 months, the mean best-corrected visual acuity, the retinal thickness of the foveal center, and the maximum retinal thickness at each scheduled visit. RESULTS The noninferiority of the 30%-dose PDT compared with the 50%-dose PDT for the primary outcomes was not demonstrated. The optical coherence tomography-based improvement rate in the 30%-dose PDT group was less than that in the 50%-dose PDT group both at 6 months (73.8% vs 92.9%; alpha = 0.0125, P = .006) and at 12 months (75.4% vs 94.6%; alpha = 0.0125, P = .004). The fluorescein angiography-based improvement rate in the 30%-dose PDT group was less than that in the 50%-dose PDT group both at 6 months (68.9% vs 91.1%; alpha = 0.0125, P = .003) and at 12 months (68.9% vs 92.9%; alpha = 0.0125, P = .001). The subretinal fluid recurrence rate in the 30%-dose PDT group was greater than that in the 50%-dose PDT group (24.0% vs 5.7% at 12 months; P = .010, determined by use of the log-rank test). The fluorescein leakage recurrent rate in the 30%-dose PDT group was significantly higher than that in the 50%-dose PDT group (16.7% vs 3.8% at 12 months; P = .03, determined by use of the log-rank test). No ocular adverse event was encountered in the study. CONCLUSIONS AND RELEVANCE A 50% dose of verteporfinmay be more effective at resolving subretinal fluid and fluorescein leakage, and with better visual outcomes, than a 30% dose for acute central serous chorioretinopathy.

Zhou, L., et al. (2021). "Subthreshold Micropulse Laser vs. Conventional Laser for Central Serous Chorioretinopathy: a Randomized Controlled Clinical Trial." Frontiers in medicine **8**.

Purpose: To investigate the effectiveness and safety of 577-nm subthreshold micropulse laser (SML) on acute central serous chorioretinopathy (CSC). Methods: One hundred and ten patients with acute CSC were randomized to receive SML or 577-nm conventional laser (CL) treatment. Optical coherence tomography and best-corrected visual acuity (BCVA) were performed before and after treatment. Results: At 3 months, the complete resolution of subretinal fluid (SRF) in 577-nm SML group (72.7%) was lower than that in CL group (89.1%) (Unadjusted RR, 0.82; P = 0.029), but it was 85.5 vs. 92.7% at 6 months (unadjusted RR, 0.92; P = 0.221). The mean LogMAR BCVA significantly improved, and the mean central foveal thickness (CFT) significantly decreased in the SML group and CL group (all P < 0.001) at 6 months. But there was no statistical difference between the two groups (all P > 0.05). In the SML group, obvious retinal pigment epithelium (RPE) damage was shown only in 3.64% at 1 month but 92.7% in the CL group (P < 0.001). Conclusions: Although 577-nm SML has a lower complete absorption of SRF compared with 577-nm CL for acute CSC at 3 months, it is similarly effective as 577-nm CL on improving retinal anatomy and function at 6 months. Importantly, 577-nm SML causes less damage to the retina.

Zhu, C. Y., et al. (2016). "Efficacy of Chinese medicine formula for central serous chorioretinopathy." International eye science **16**(5): 916-919.

AIM:To investigate the clinical efficacy of Chinese medicine formula for macular edema in the patients with phlegm intrinsic type and central serous chorioretinopathy (CSCR). METHODS:With randomized controlled method, 56 cases (71 eyes) with phlegm intrinsic type and CSCR were randomly divided into the control group and the treatment group. The control group included 28 cases (35 eyes), and the treatment group 28 cases(36 eyes). The patients in the control group received injections of 2mL compound anisodine beside superficial temporal artery, once a day. Based the above treatment, the patients in the treatment group were given 10g oral Chinese medicine formula for macular edema, twice a day. After 3mo, the overall curative effect, visual acuity, visual field mean sensitivity and the OCT in both groups were measured and evaluated. RESULTS:After treatment, the total effective rate in treatment group was 89%, which was significantly higher than 79% in the control group (P<0.05).Compared with the control group, the vision and visual field mean sensitivity in the treatment group significantly increased, and macular center thickness of 1mm diameter range, the average thickness of the macular area of 6mm diameter and the diameter and the central area and the total macular volume were statistically significantly lower (P<0.05). CONCLUSION:Chinese medicine formula for macular edema may ameliorate the visual acuity and visual field in the patients with phlegm intrinsic type and central serous chorioretinopathy, reducing edema lesions and promoting the recovery of visual function.
